# Selective Enrichment of Nitrososphaera viennensis-Like Ammonia-Oxidizing Archaea over Ammonia-Oxidizing Bacteria from Drinking Water Biofilms

**DOI:** 10.1128/spectrum.01845-22

**Published:** 2022-11-29

**Authors:** Yissue Woo, Mercedes Cecilia Cruz, Stefan Wuertz

**Affiliations:** a Singapore Centre for Environmental Life Sciences Engineering, Nanyang Technological University, Singapore, Singapore; b Singapore Centre for Environmental Life Sciences Engineering, Interdisciplinary Graduate Programme, Nanyang Technological University, Singapore, Singapore; c School of Civil and Environmental Engineering, Nanyang Technological University, Singapore, Singapore; Oklahoma State University

**Keywords:** drinking water, ammonia oxidation, archaea, monochloramine, selective enrichment

## Abstract

Ammonia-oxidizing archaea (AOA) can oxidize ammonia to nitrite for energy gain. They have been detected in chloraminated drinking water distribution systems (DWDS) along with the more common ammonia-oxidizing bacteria (AOB) and nitrite-oxidizing bacteria (NOB). To date, no members of the AOA have been isolated or enriched from drinking water environments. To begin the investigation of the role of AOA in chloraminated DWDS, we developed a selective approach using biofilm samples from a full-scale operational network as inoculum. A Nitrososphaera viennensis-like AOA taxon was enriched from a mixed community that also included Nitrosomonas-like AOB while gradually scaling up the culture volume. Dimethylthiourea (DMTU) and pyruvate at 100 μM were added to promote the growth of AOA while inhibiting AOB. This resulted in the eventual washout of AOB, while NOB were absent after 2 or 3 rounds of amendment with 24 μM sodium azide. The relative abundance of AOA in the enrichment increased from 0.2% to 39.5% after adding DMTU and pyruvate, and further to 51.6% after filtration through a 0.45-μm pore size membrane, within a period of approximately 6 months.

**IMPORTANCE** Chloramination has been known to increase the risk of nitrification episodes in DWDS due to the presence of ammonia-oxidizing microorganisms. Among them, AOB are more frequently detected than AOA. All publicly available cultures of AOA have been isolated from soil, marine or surface water environments, meaning they are allochthonous to DWDS. Hence, monochloramine exposure studies involving these strains may not accurately reflect their role in DWDS. The described method allows for the rapid enrichment of autochthonous AOA from drinking water nitrifying communities. The high relative abundance of AOA in the resulting enrichment culture reduces any confounding effects of co-existing heterotrophic bacteria when investigating the response of AOA to varied levels of monochloramine in drinking water.

## INTRODUCTION

Secondary disinfection is practiced by water utilities to increase the biological safety of the distributed treated drinking water, by discouraging the growth of contaminating organisms introduced by ingress (1) and, thus, guarantees the water biostability. Monochloramine replaced chlorine as the choice disinfectant in certain countries practicing secondary disinfection like Singapore (Environmental Public Health [Quality of Piped Drinking Water] Regulations 2008 [Cap 95, 2002 Rev Ed]) and the USA (2), because the combined chlorine is less reactive than free chlorine. This reduces the formation of disinfection by-products in the water that are potentially carcinogenic or genotoxic (3–7), and favors the persistence of disinfectant in drinking water distribution systems (DWDS) compared to that of free chlorine.

However, abiotic decay of monochloramine and reactions between monochloramine and organic matter release ammonia that may be oxidized by ammonia-oxidizing microorganisms (AOM) as an energy source (8–11). Nitrification is a common occurrence in DWDS that practice secondary disinfection with monochloramine and is associated with increased heterotrophic plate counts (12–14). Since nitrifying microorganisms are often autotrophic organisms that fix inorganic carbon from the environment into biomass, biodegradable dissolved organic carbon will be reintroduced into DWDS when regrowth of nitrifiers occur, which can facilitate the regrowth of heterotrophic microorganisms, including potential pathogens (15–17). In addition, nitrite produced by ammonia oxidation can readily react with monochloramine to further release ammonia, while accelerating the monochloramine decay rate in the DWDS (8, 10), further supporting the growth of AOM, and result in a feedback loop of AOM growth and monochloramine decay.

In DWDS experiencing nitrification episodes, ammonia-oxidizing bacteria (AOB) and nitrite-oxidizing bacteria (NOB) have been commonly detected in the bulk water (13, 18–22), unlike ammonia-oxidizing archaea (AOA), which were reported at ammonia concentrations near or below detection limits (21, 23–26). The detection of nitrification by AOA in DWDS may be masked by the slower rate of ammonia oxidation by AOA, which may not be sufficient to trigger detectable nitrification episodes unless during long periods of water stagnation. Therefore, the present chloramine studies have focused on AOB, and the efficiency of disinfection on AOA is currently uninvestigated (27–31).

Those AOA in DWDS identified at the genus level by sequence analysis of the archaeal ammonia monooxygenase subunit A (*amoA*) gene were associated with the Group 1.1a *Nitrosopumilus* cluster belonging to the *Candidatus* Nitrosopumilus order (21, 23, 25, 26), which contain mostly marine AOA (32). Using sequence analysis of the 16S rRNA V4-V5 hypervariable region, one study reported AOA in a biofilm as being associated with the Marine Group 1 Thaumarchaeota (24). In Singapore, AOA have been detected together with NOB in the DWDS biofilms, in sections of the distribution network with lower monochloramine concentrations, whereas in sections with higher monochloramine concentrations, AOB were often the only AOM detected (24). Additionally, AOA were not detected in biofilms growing on sensors installed in the DWDS (22).

To date, the enrichment or isolation of AOA from DWDS has not been reported. The cultivation of AOA is more challenging than that of AOB because of their slower growth rate, which can ultimately lead to the culture being overgrown by AOB (33–35). In this study, a novel method for selective enrichment of AOA was developed to address the above challenges, to remove any coculturing AOB and stimulate the growth of AOA without relying on the use of filtration or antibiotic treatment. This new method should allow for a more rapid enrichment of AOA from freshwater environments for subsequent physiological studies by retaining the ability to remove hydrogen peroxide, which is provided *in situ* by microbial communities producing catalases and/or peroxidases, in the culture.

## RESULTS

### Enrichment strategy for AOA from low-biomass drinking water environments.

Nitrososphaera viennensis-like AOA were selectively enriched over Nitrosomonas oligotropha-like AOB to a relative abundance of over 50% as measured by 16S rRNA gene metabarcoding. This was achieved through the combined use of dimethylthiourea (DMTU) to inhibit AOB growth, and pyruvate to stimulate AOA growth, and filtration through a 0.45-μm pore size filter membrane to further improve the purity of the AOA enrichment.

Initially, biofilm samples from a full-scale DWDS known to experience nitrification events were used as inoculum for the enrichment of nitrifiers. Those samples were collected in a survey study from 21 sections cut out from buried water distribution pipes at diverse locations in a tropical city (24), involving pipes with diameters from 100 mm to 700 mm, with pipe ages ranging from 6 years to 60 years, and most of the pipes being cement lined. The pipe materials included cast iron, ductile iron, and stainless steel, and the pipe cutouts included straight and bent-connector sections.

**FIG 1:**
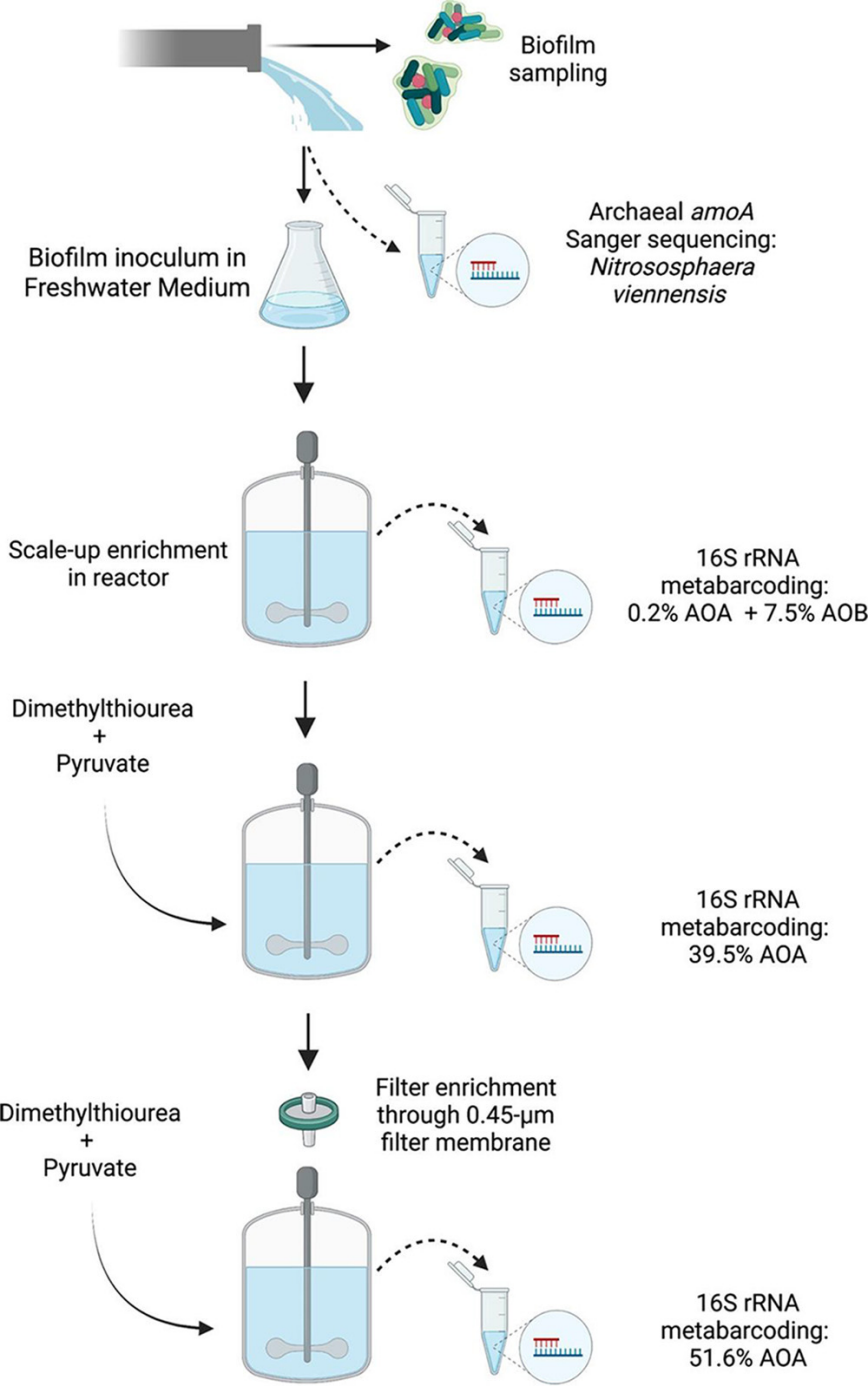
Strategy to enrich Ammonia-Oxidizing Archaea (AOA) from drinking water biofilms.

The initial steps were done in 100-mL static cultures before scaling up to 1.5-L stirred and aerated culture vessels, with the enrichment culture maintained at 30°C to mimic the average water temperature of the full-scale DWDS in Singapore. Both 16S rRNA gene metabarcoding and the quantification of bacterial and archaeal *amoA* gene copies by qPCR were used to determine the purity of the AOA enrichment (Fig. 1).

AOA were outgrown by AOB during early enrichment efforts, but the enrichment improved after the addition of dimethylthiourea (DMTU) to inhibit AOB growth and pyruvate to stimulate AOA growth. The purity of the AOA enrichment was reexamined with 16S rRNA gene metabarcoding and AOB were found to have been washed out to below detection. The culture was filtered through a 0.45-μm pore size filter membrane to further improve the purity of the AOA enrichment. The strategy for selective enrichment of AOA over AOB using DMTU and pyruvate was then validated in a separate experiment.

### Taxonomy of AOA in environmental biofilm samples.

Phylogenetic analysis of the cloned archaeal 16S rRNA gene sequences (accession no. OP493080 to OP493089) revealed the AOA to be closely related to Nitrososphaera viennensis strain EN76 (Fig. S1); similarly, the nucleotide sequences of the cloned archaeal *amoA* gene fragment (accession no. OP556316 to OP556329) were closely related to Nitrososphaera viennensis strain EN76 (Fig. S2). The translated peptide sequence of the cloned archaeal *amoA* gene fragment was unique and diversely related to the genus Nitrososphaera (Fig. S3). Based on the available evidence the AOA in the enrichment were categorized as Nitrososphaera viennensis-like organisms.

### Taxonomy of AOB in environmental biofilm samples.

Phylogenetic analysis of the 16S rRNA gene amplicon sequence variants (accession no. OP550269 to OP550275) revealed the AOB to be closely related to the N. oligotropha group (Fig. S4). However, the nucleotide and translated peptide sequences of the cloned bacterial *amoA* gene fragment (accession no. OP556330 to OP556334) were diversely related to the AOB genera Nitrosomonas, Nitrospira, and Nitrosococcus (Fig. S5 and S6), with most of the clones more closely related to the N. oligotropha lineage. Based on the available evidence the AOB in the enrichment were categorized as N. oligotropha-like organisms.

### The 16S rRNA gene metabarcoding of enrichment before and after addition of DMTU and pyruvate.

The relative sequence abundance of AOA “*Candidatus*_Nitrososphaera” in the enrichment increased to 39.5% after continuous culturing with DMTU and pyruvate, from the initial relative sequence abundance of 0.2% (Table 1). The initial enrichment was dominated by the phylum *Proteobacteria*, with the AOB Nitrosomonas at a relative abundance of 7.5% as determined by 16S rRNA gene metabarcoding, while Hyphomicrobium was the most abundant taxon at 33.1% (Fig. S9). After continuous culturing with DMTU and pyruvate, involving 6 transfers, the community had changed. At 39.5% relative abundance, the most abundant phylum in the enrichment belonged to the AOA Thaumarchaeota, followed by Bacteroidetes at 32.7% (Fig. S10).

**TABLE 1:** Effect of filtration and addition of NN’-dimethylthiourea and pyruvate on enrichment of ammonia oxidizing archaea from drinking water biofilms

	Nitrifier relative abundance (%)^*a*^		
Treatment	*Nitrososphaera* (AOA^*b*^)	*Nitrosomonas* (AOB^*c*^)	Non-nitrifiers
None	0.20	7.50	92.3
DMTU^*d*^, pyruvate	39.5	ND^*e*^	60.5
Filtration, DMTU, pyruvate	51.6	ND	46.3

a Based on 16S rRNA gene metabarcoding.

b Ammonia oxidizing archaea.

c Ammonia oxidizing bacteria.

d NN’-Dimethylthiourea.

e Not detected.

### Validation of enrichment strategy: washout of AOB during AOA enrichment.

To confirm that the washout of AOB from the AOA enrichment was indeed the consequence of the enrichment strategy, AOA and AOB enrichments were diluted to approximately 10^3^ *amoA* gene copies/mL and mixed in approximately equal proportions, to compare the enrichment strategy with variations of the method. The experiment was performed with 3 groups: (i) With addition of DMTU and pyruvate, (ii) with addition of pyruvate only, and (iii) without addition of DMTU and pyruvate. Each group consisted of 3 replicates, and the experiment was conducted for a duration that covered 3 subsequent subculture events, which occurred when the supplied concentration of ammonium substrate was depleted.

Droplet digital PCR (ddPCR) was used to quantify AOA and AOB using an assay based on newly designed *Nitrososphaera amoA* primers and *Nitrosomonas amoA* primers, respectively (Table 1). The specificity of the primers was confirmed in qPCR experiments, where the *Nitrososphaera amoA* primers were challenged with 10^7^ copies of *Nitrosomonas amoA* plasmids per reaction, and *Nitrosomonas amoA* primers were challenged with 10^7^ copies of *Nitrososphaera amoA* plasmids per reaction. There was no evidence of cross-reaction of the newly designed primers. Non-nitrifying bacteria were not quantified because the experiment focused on the elimination of AOB from the enrichment culture.

**FIG 2:**
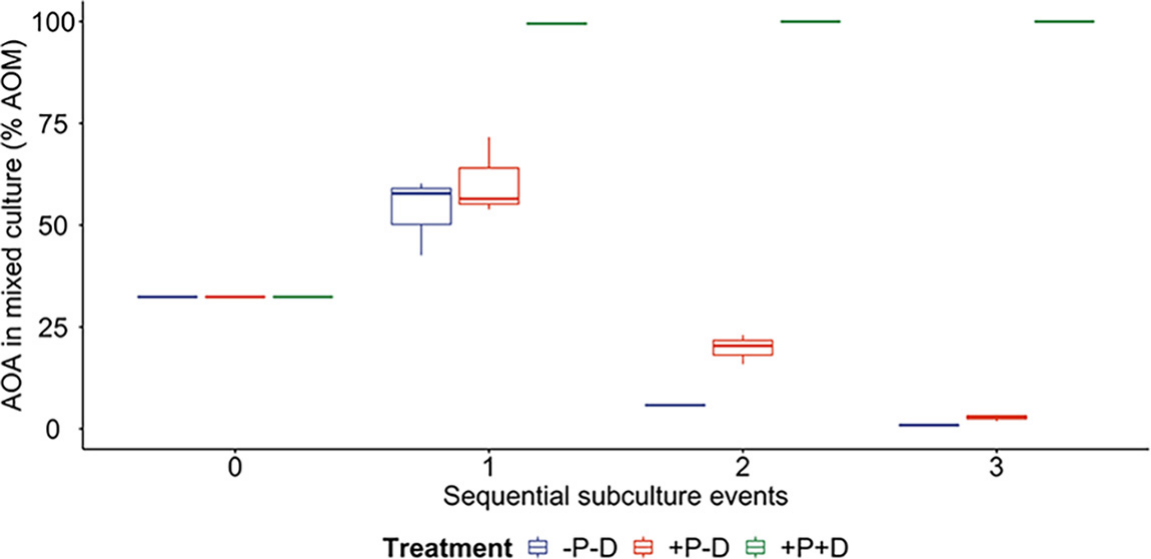
Temporal changes in the proportion of AOA in an AOA-AOB mixed culture initially containing equal amounts of AOA and AOB plus non-nitrifiers. Separate AOA and AOB cultures enriched from drinking water biofilms were diluted to approximately 10^3^ *amoA* gene copies/mL and mixed in equal proportions to validate the AOA enrichment strategy. The proportion of ammonia oxidizing microorganisms (AOM) that were AOA increased over time to 99.5%, and eventually to 99.9%, from the initial 32.4%, as measured by ddPCR. *N *= 3, error bars represent SD. *P* = pyruvate; D = DMTU. Green, cultures with DMTU and pyruvate; orange, cultures with pyruvate only; and blue, cultures without DMTU or pyruvate. Subculture event 0 refers to the starter culture, and subculture events 1, 2, and 3 were carried out when approximately 90% of supplied ammonium had been depleted. The time required to reach depletion changed depending on the proportion of AOA in the culture.

Upon completion of 3 subculture events, the proportion of AOA in the AOA + AOB mixture increased from 32.4% to 99.9% when cultured in the presence of 100 μM DMTU and pyruvate (+P+D) each. In the cultures supplied with only 100 μM pyruvate (+P -D), the proportion of AOA increased to 60.7%, while in the cultures that did not receive DMTU and pyruvate (-P -D), the proportion of AOA increased to 53.5% before the first subculture. However, after 3 subculture events, the AOA had been outcompeted by AOB except for those cultures supplied with 100 μM DMTU and pyruvate (Fig. 2).

### Using 16S rRNA gene metabarcoding on enrichment with filtration and addition of DMTU and pyruvate.

The relative abundance of the AOA member “*Candidatus*_Nitrososphaera” in the enrichment increased to 51.6% following the filtration of the inoculum with 0.45 μm membrane filter and continuous culturing with DMTU and pyruvate, up from a relative abundance of 39.5% before filtration was introduced (Table 1). After filtration and continuous culturing with DMTU and pyruvate, the second most abundant phylum after Thaumarchaeota was Proteobacteria at 36.7% relative abundance, with Hyphomicrobium being the most abundant Proteobacteria genus at 12% (Fig. S11). This was achieved after 2 transfers using a filtered inoculum.

### Fluorescence *in-situ* hybridization and microscopic observation of enriched AOA and AOB.

**FIG 3:**
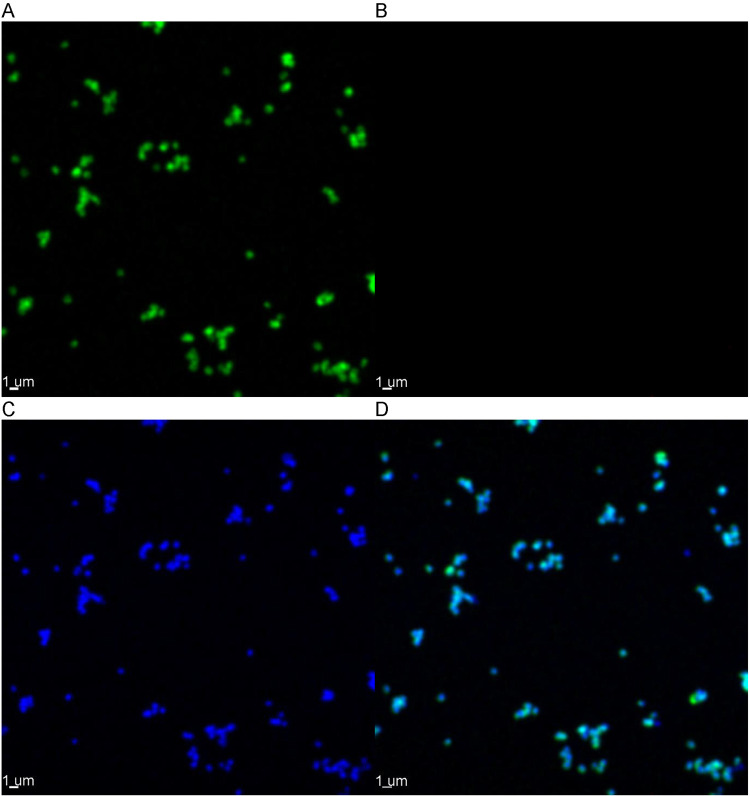
Fluorescence *in situ* hybridization of the AOA enrichments observed with confocal laser scanning microscopy under a 100× objective with oil immersion. The cells shown in green were hybridized with the Archaeal 16S rRNA probes (A); red cells (none visible) were hybridized with AOB 16S rRNA probes (B); and blue cells were stained with DAPI (C). The final panel displays the superimposed fluorescence signals (D). The scale bar represents 1 μm.

**FIG 4:**
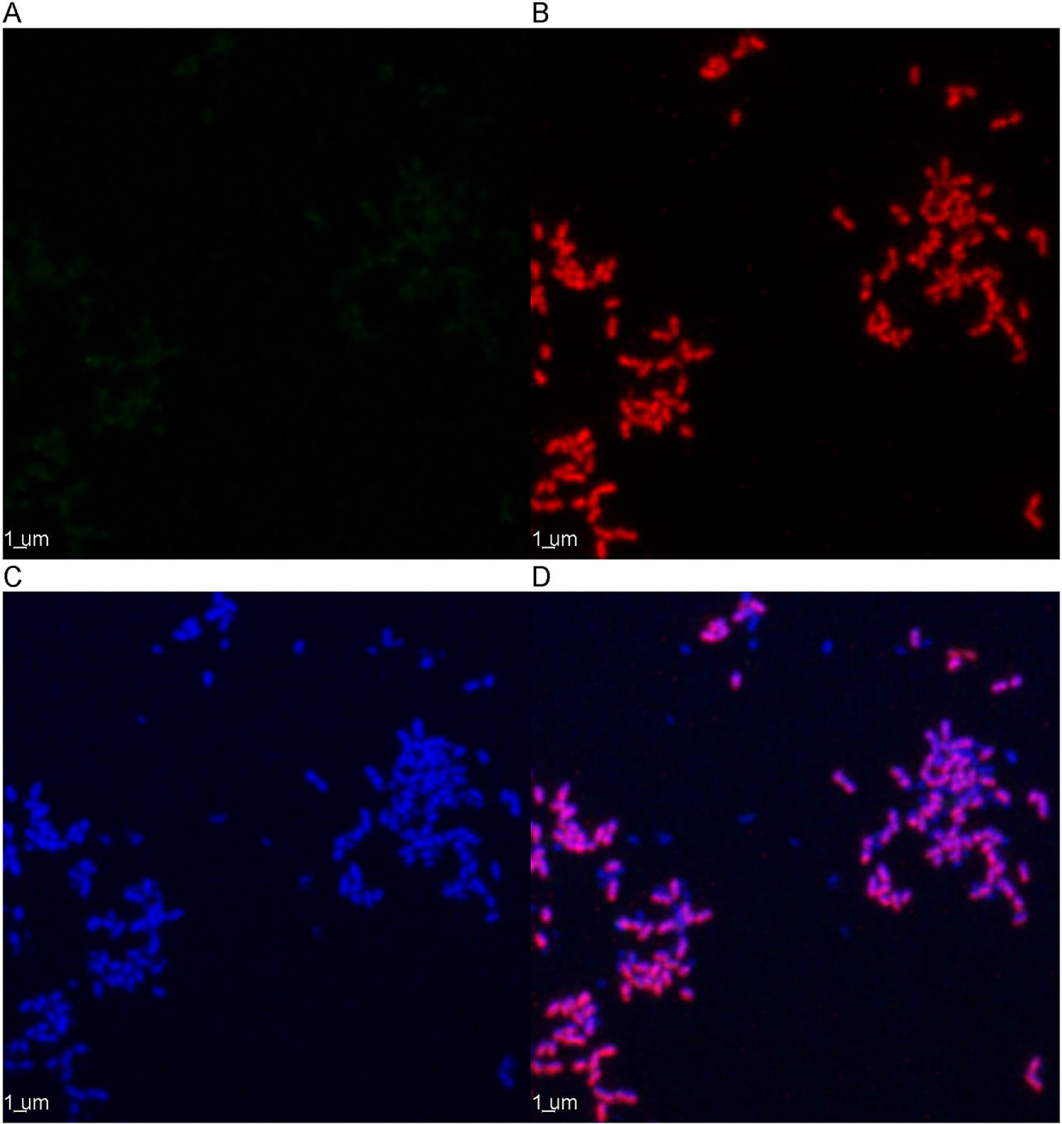
Fluorescence *in situ* hybridization of the AOB enrichments observed with confocal laser scanning microscopy under a 100x objective with oil immersion. The cells shown in green (none visible) were hybridized with the Archaeal 16S rRNA probes (A); red cells were hybridized with AOB 16S rRNA probes (B); and blue cells were stained with DAPI (C). The final panel displays the superimposed fluorescence signals (D). The scale bar represents 1 μm.

AOA enrichment cultures had cells with irregular spherical morphologies and diameters <0.5 μm (Fig. 3 and Fig. S13), while AOB cells were not observed. AOB enrichment cultures had short rod-shaped cells with lengths of 0.5 to 1 μm, while AOA cells were not observed (Fig. 4 and Fig. S14). These morphologies resemble the published morphologies of Nitrososphaera viennensis and Nitrosomonas spp.

## DISCUSSION

In this study, the supplementation of the enrichment culture with DMTU and pyruvate enhanced AOA growth and inhibited AOB, based on their increased sensitivity to thiourea inhibition. After successive rounds of culturing with DMTU and pyruvate, AOB were reduced to below detection level, while the relative abundance of AOA improved greatly to 39.5% (Table 2). To further improve the enrichment of AOA, filtration through a 0.45-μm membrane filter was adopted with the continued use of DMTU and pyruvate, which increased the AOA relative abundance to 51.6% (Table 2). This progress was achieved within 6 months, after the adoption of FWM as the culture medium for the enrichment of AOA.

**TABLE 2:** Primers used in this study

Primer	Sequence (5′–3′)	Target	Source
Arch21F	TTCCGGTTGATCCYGCCGGA^*a*^	Archaea 16S	74
1492R	GGTTACCTTGTTACGACTT^*a*^	Archaea and Bacteria 16S	75
Arch_amoAF	STAATGGTCTGGCTTAGACG	Archaeal *amoA*	72
Arch_amoAR	GCGGCCATCCATCTGTATGT		
amoA332F	GGGGTTTCTACTGGTGGT	Bacterial *amoA*	73
amoA822R	CCCCTCKGSAAAGCCTTCTTC		
NSS_amoAF	CGCTGCTAACCATCAACGCA	*Nitrososphaera amoA*	This study
	ACACTGCTAACCATCAACGCAG		
NSS_amoAR	GCACCCACAGCGAGCAT		
	GCGCCCACTACGAGCATTG		
NSM_amoAF	TATGTTCGCCTGATTGAGCAAGG	*Nitrosomonas amoA*	This study
	TACGTTCGTTTGATTGAACAAGG		
NSM_amoAR	CCACCATACGCAGAACATCAGCAT		
	CCACCATACACAGAACATCAGCAT		
515F	GTGYCAGCMGCCGCGGTAA^*b*^	16S rRNA^*a*^	76
926R	CCGYCAATTYMTTTRAGTTT^*c*^		
M13 Forward (-20)	GTAAAACGACGGCCAG	Vector insert	Invitrogen
M13 Reverse	CAGGAAACAGCTATGAC		

a V4-V5 hypervariable region.

b Adapter: 5′-TCGTCGGCAGCGTCAGATGTGTATAAGAGACAG-3′.

c Adapter: 5′-GTCTCGTGGGCTCGGAGATGTGTATAAGAGACAG-3′.

In the validation experiment, AOB washout was again observed in the treatment group with DMTU and pyruvate (+P+D). When DMTU was absent in the culture medium (+P -D) (-P -D), AOA initially outcompeted AOB, but were subsequently replaced by AOB after subculturing. The initial advantage of AOA could be due to their higher growth yield per unit of ammonia oxidized. The Calvin-Benson-Bassham (CBB) cycle used by AOB to assimilate bicarbonate is less efficient than the 3-hydroxypropionate/4-hydroxybutyrate (HP/HB) cycle used by AOA (15, 36). It is possible that in the first culture period, the few AOB cells present in the culture failed to compete with AOA for ammonia, which allowed AOA to proliferate. After subculturing in the absence of DMTU, the increased number of AOB cells in the culture would be sufficient to effectively compete with AOA for ammonia, leading to the eventual washout of AOA after just 2 subcultures. The incubation experiment was conducted at ambient temperature on a benchtop orbital shaker.

Few strategies for the enrichment of AOA have been reported, with most referring to marine environments (37–41) and some to soil (42), wastewater (43), and hot springs (44). The AOA enriched from biofilm samples in this study were categorized as Nitrososphaera viennensis-like, a mesophilic species first isolated from soil (42); other Nitrososphaera spp. have been found in freshwater (45) and wastewater (46). AOA can grow at extremely low concentrations of ammonia compared to AOB (35). The maximum growth rate of AOA is lower than that of AOB, and when they are present together at higher concentrations of ammonia, AOA will be overgrown by AOB (33–35). The AOB enrichment yielded taxa closely related to N. oligotropha, which have a reported *K_s_* value of ammonia oxidation of 3.6 μM, and therefore would be growing at their maximum growth rate with the 2 mM ammonium supplied (33–35).

Previous approaches employing filtration through a 0.45 μm pore size filter and/or antibiotic treatments met with varying degrees of success, with filtration usually more successful, yet at times sterilizing the culture (41, 47–49). Others reported that antibiotic treatment slowed the growth of AOA in the enrichment culture, likely due to the removal of catalase-producing microorganisms from the culture, therefore subjecting the AOA to growth inhibition by hydrogen peroxide (50, 51).

In the early enrichment efforts, when AOB were observed to outgrow AOA in the enrichment culture, filtering the culture through a 0.45 μm membrane filter or treating the culture with antibiotics resulted in the failure of the enrichment, where ammonia oxidation was no longer detected. It is possible that low AOA biomass present in the culture, and the application of antibiotics considerably slowed the growth of the AOA. In addition, the previously described methods involved larger quantities of sediment samples, which potentially contained more AOA cells. In contrast, the biofilm samples obtained from the DWDS were of limited quantity, and fewer AOA cells were present because of the presence of monochloramine as secondary disinfectant. The loss of catalase and/or peroxidase activity from the enrichment culture was suspected to contribute to the early failure of the AOA enrichment.

Although the cell size of Nitrososphaera viennensis is smaller than that of N. oligotropha, the size of the AOA (0.78 ± 0.13 μm diameter) is slightly larger than the 0.45 μm pores of the filters used, which could explain why initial attempts to select for AOA with filtration failed (33–35). The non-rigid nature of microbial cells should allow some of the AOA cells to pass through the filter, with the larger AOB and heterotrophic cells staying behind. Microscopic observation of the enriched AOA stained with fluorescence *in situ* hybridization (FISH) revealed that the archaeal cells have diameters smaller than 0.5 μm, and the enriched AOB stained with FISH were confirmed to be larger with lengths of approximately 1 μm. Increasing the number of AOA cells present before filtration should increase the likelihood of finding some in the filtrate.

Hydrogen peroxide and other radical oxygen species (ROS) are by-products produced during biological aerobic metabolism, and are generally toxic to cells at high concentrations, introducing oxidative damage to DNA and cellular components (52). Thiourea, while being a hydroxyl radical scavenger (53), also acts a metal chelator, allowing it to be a nonspecific hydroxyl radical scavenger. The chelation of copper ion makes allylthiourea (ATU) a potent inhibitor of ammonia oxidation in AOB (50), but less so in AOA (54). DMTU, in addition to being a hydroxyl radical scavenger, also acts as a hydrogen peroxide scavenger (55), and, hence, is a suitable agent for removing hydrogen peroxide from the culture. *α*-keto acids like pyruvate are known to aid the growth of the AOA Nitrososphaera, perhaps by enabling mixotrophy (33). In a study to determine how *α*-keto acids help AOA grow, it was found that they served as hydrogen peroxide scavengers, as the effects were replicated when the *α*-keto acids were substituted with DMTU (51). Therefore, using 100 μM DMTU and pyruvate allowed AOA to grow without having to rely on catalase-producing heterotrophic microorganisms (51).

The method described in this study allows for the selective enrichment of AOA over AOB without an initial filtration step or antibiotic treatment to remove AOB from the culture. Hence, the presence of some heterotrophs producing enough catalases or peroxidases prevented inhibition of AOA by hydrogen peroxide. Upon selective enrichment of AOA cells, multiple filtration steps were introduced to physically remove non-AOA cells.

Previous studies involving chloramination and nitrification had focused on pure cultures of AOB that are allochthonous to drinking water environments (27, 29, 56, 57), or on mixed-nitrifier biofilms that were often uncharacterized or cultivated from water treatment plant filters (9, 23, 28, 58–60). Obtaining enriched AOA cultures that are autochthonous to drinking water environments will allow for more accurate studies of nitrifying activities of AOA in DWDS resulting from secondary disinfection with chloramine.

In conclusion, this novel strategy for enriching AOA from DWDS and other ecological niches should facilitate studies by shortening the duration of the enrichment period needed to produce sufficient AOA biomass from years (42) to months and ease the isolation of AOA for detailed characterization.

## MATERIALS AND METHODS

### Culturing conditions for *Nitrososphaera viennensis*-like AOA and *N. oligotropha*-like AOB.

The identity of the AOA was determined through sequence analysis of cloned 16S rRNA and archaeal *amoA* gene fragments, which revealed Nitrososphaera viennensis as the most probable organism (Fig. S1 and S3). Hence, Freshwater Medium (FWM) was chosen as growth medium (33). FWM was adapted from M. Kerou and C. Schleper (33) by replacing the vitamin solution with the one used by W. E. Balch et al. (61). The composition of FWM was: 1 g/L NaCl, 0.4 g/L MgCl_2_.6H_2_O, 0.1 g/L CaCl_2_.2H_2_O, 0.2 g/L KH_2_PO_4_, 0.5 g/L KCl, 1 mL/L trace element solution (Per L: 100 mg MnCl_2_.4H_2_O, 30 mg H_3_BO_3_, 36 mg Na_2_MoO_4_.2H_2_O, 2 mg CuCl_2_.2H_2_O, 24 mg NiCl_2_.6H_2_O, 190 mg CoCl_2_.6H_2_O, 144 mg ZnSO_4_.7H_2_O), 1 mL/L vitamin solution, and 7.5 μM ferric sodium EDTA. The pH of the FWM was adjusted to pH 8.0 and buffered with HEPES solution (10 mM HEPES, 6 mM NaOH). For energy, AOA and AOB enrichment cultures received 2 mM and 5 mM NH_4_Cl, respectively, and 2 mM NaHCO_3_ was supplied as a carbon source. The FWM was further supplemented with 100 μM DMTU and sodium pyruvate to stimulate the growth of the AOA.

Enrichment cultures were inoculated with biofilms identified to be rich in AOA or AOB (24) and grown in 50 mL of FWM, containing 2 or 5 mM NH_4_Cl and 24 μM sodium azide to inhibit the growth of NOB, in 100-mL wide-neck Erlenmeyer flasks under static conditions in a 30°C incubator. The cultures were subcultured upon depletion of 90% of the ammonia supplied by inoculating 10% of the spent culture into fresh media containing 2 or 5 mM NH_4_Cl. Twenty-four micromolar sodium azide was continuously supplied until accumulation of nitrite was consistently observed and confirmation that NOB were undetectable by quantitative PCR (qPCR).

To wash out coculturing AOB from the AOA enrichment culture, 100 μM DMTU and sodium pyruvate was added to inhibit the growth of AOB and to stimulate the growth of AOA. DMTU and sodium pyruvate were constantly supplied, even upon confirmation that AOB were undetectable by qPCR.

The initial enrichments were diluted 10x into 100 mL of FWM in a sterile 250-mL Erlenmeyer flask with a magnetic stirrer rod, supplemented with 4 mM NH_4_Cl, and mixed in the dark at room temperature on a magnetic stirrer at approximately 100 rpm. Upon depletion of the supplied NH_4_Cl, 150 mL of the spent stirred culture was diluted into 1.5 L of FWM in a stirring culture vessel (Nalgene Polycarbonate Magnetic Culture Vessel) (Fig. S12). The cultures were stirred at low speed using a magnetic stirrer, aerated with filtered air at 1 L/min, and incubated at 30°C using a silicon heating jacket with a temperature controller. The cultures were monitored via a sampling port for substrate utilization and growth of the nitrifiers. When 90% of the supplied ammonia or nitrite had been depleted, 500 mL of the spent culture was collected for storage at 4°C and the remaining volume wasted. The culture vessels were then scrubbed with Virkon S solution using dishwashing brushes to remove biofilms and then rinsed with ultrapure Type 1 water until foaming was no longer observed. Next 15 mL of the spent culture was inoculated into 1.5 L of FWM in the cleaned culture vessel. For every subculture event, the inoculum was filtered through a 0.45-μm filter membrane.

Subculturing of the AOA and AOB enrichments was performed by diluting 1% of spent culture into fresh media, before supplying the cultures with 2 mM and 5 mM NH4Cl, respectively. The AOB culture required 3 days and the unfiltered AOA culture 14 days to deplete 5 mM ammonium and 2 mM ammonium, respectively. When the AOA inoculum was filtered first a longer incubation period of 28 days was necessary.

### Substrate concentration measurement.

Ammonia concentrations were determined using the EPA Hach Method 10205 and Ammonia (Nitrogen) Test 2Strips 0–6.0 mg/L (HACH Co.), while nitrite concentrations were determined using the HACH method 10207 and 10237, and the Nitrate and Nitrite Test Strips (HACH Co.). The HACH methods were performed using the DR3900 Laboratory Spectrophotometer for water analysis (HACH Co.).

### Cloning and sequencing of archaeal 16S rRNA and *amoA* genes.

Archaeal 16S rRNA gene was amplified from nucleic acid extracted from biofilm samples obtained from the DWDS, using the archaeon-specific primers listed in Table 2, in a PCR comprised of 0.2 μL of BioReady *Taq* DNA polymerase, 2.5 μL of 10× buffer with 15 mM MgCl_2_, 1 μL of 25 mM MgCl_2_, 1 μL of 10 mM dNTP mix, 0.5 μL of forward and reverse primer, 14.3 μL of nuclease-free water, and 5 μL of template DNA in a 25 μL reaction volume. The PCR was performed with the following cycle conditions: 5 min at 94°C, followed by 25 cycles of 30 s at 94°C; 30 s at 55°C; 90 s at 72°C; and 5 min at 72°C. The final cycle ended with infinite hold at 4°C.

Similarly, the archaeal *amoA* gene was amplified using the Arch_amoA primers listed in Table 2, in a PCR comprised of 12.5 μL of AmpliTaq Gold 360 mastermix (Applied Biosystems), 200 nM forward and reverse primer, and 5 μL of template DNA in a 25 μL reaction volume. The PCR was performed with the following cycle conditions: 5 min at 95°C, followed by 35 cycles of 30 s at 95°C; 60 s at 62°C; 60 s at 72°C; and 5 min at 72°C. The final cycle ended with infinite hold at 4°C.

The bacterial *amoA* gene was also amplified from those nucleic acid extracts, using the amoA332F and amoA822R primers listed in Table 2, in a PCR comprised of 12.5 μL of AmpliTaq Gold 360 mastermix (Applied Biosystems), 200 nM forward and reverse primer, and 5 μL of template DNA in a 25 μL reaction volume. The PCR was performed with the cycling parameter: 5 min at 95°C, followed by 35 cycles of 30 s at 95°C, 60 s at 62°C then 60 s at 72°C, followed by 5 min at 72°C, and ending with an infinite hold at 4°C.

The amplicons were immediately cloned into plasmids using the TOPO TA Cloning Kit for Sequencing, with One Shot TOP10 Chemically Competent Escherichia coli (Invitrogen), according to the manufacturer’s instructions. Between 10 and 20 individual plasmid clones were extracted for plasmids using the PureLink Quick Plasmid Miniprep Kit (Invitrogen), according to the manufacturer’s instructions. The plasmids were screened using M13 primers supplied by the cloning kit. Amplicons were generated in 4 replicates from the positive clones, then pooled and purified using the PureLink PCR purification kit (Invitrogen). The purified amplicons were sent to 1st BASE for Sanger sequencing using the M13 Forward (-20) primer. The PCR comprised of 12.5 μL of AmpliTaq Gold 360 mastermix (Applied Biosystems), 200 nM forward and reverse primer, and 5 μL of template DNA in a 25 μL reaction volume. The PCR was performed with the following cycle conditions: 5 min at 95°C, followed by 35 cycles of 30 s at 95°C; 30 s at 55°C; 60 s at 72°C; and 5 min at 72°C. The final cycle ended with an infinite hold at 4°C.

### Sequence analysis of archaeal and bacteria 16S rRNA, and *amoA* genes.

The sequence data were read with SnapGene Viewer (SnapGene software from GSL Biotech; available at snapgene.com). A random clone *amoA* nucleotide sequence was used to translate to the *amoA* protein sequence, using Frame 1 output of the EMBOSS Transeq tool (62). The translated protein sequence was then used in a pBLAST (63) search, against the non-redundant protein database, to obtain a candidate list of organisms, with a percentage identity cutoff at 90%. The list of translated *amoA* sequences of the clones and the *amoA* peptide sequences of the candidate organisms were compiled into a FASTA file. Alternatively, the random clone *amoA*, archaeal 16S rRNA gene nucleotide sequence or 16S rRNA gene amplicon sequence variant from metabarcoding was used in a BLAST (64) search against the non-redundant nucleotide database to obtain a candidate list of organisms, with a percentage identity cutoff at 80 to 90%. Similarly, the list of 16S rRNA gene and *amoA* sequences of the clones and the 16S rRNA and *amoA* nucleotide sequences of the candidate organisms were compiled into their respective FASTA files.

Evolutionary analyses were conducted in MEGA X (65, 66). In brief, the multiple sequence alignment was performed on the sequences in the FASTA files, using the ClustalW (1.6) algorithm on the nucleotide sequences and the MUSCLE algorithm on the peptide sequences, and then the alignment was exported into a .meg file. The resultant file was used to find the best protein/DNA model, using the maximum likelihood method. The best nucleotide or amino acid substitution model was chosen for the construction of the maximum likelihood tree, using the Bootstrap method for testing the phylogeny with 500 replications. The nearest-neighbor-interchange was chosen for the Heuristic method, while the default option (NJ/BioNJ) was chosen for making the initial tree.

### The 16S rRNA gene metabarcoding.

The PCR was composed of 12.5 μL of 2× KAPA HiFi HotStart ReadyMix (Kapa Biosystems), 200 nM 515F and 926R primers (Table 2), and 12.5 ng of template DNA in a 25 μL reaction volume. The PCR was performed with the following cycle conditions: 3 min at 95°C, followed by 25 cycles of 30 s at 95°C, 30 s at 55°C then 30 s at 72°C; and 5 min at 72°C. The final cycle ended with infinite hold at 4°C. Each reaction was generated in triplicates, then pooled and purified with SPRISelect magnetic beads (Beckman Coulter) with double size selection ratio of 0.85 to 0.56, according to the manufacturer’s instructions. The quality of the amplicons was validated with the Agilent 4200 TapeStation System, using the AgilentD1000 ScreenTape Assay (Agilent Technologies, Inc). Sequencing was performed with MiSeq Illumina 2 × 300 bp chemistry, and the sequence reads were analyzed through the DADA2 version 1.10.1 pipeline (67, 68), performed in R version 3.5.3 (69) using Mac OS version 10.14.4.

### Designing primers for quantification in ddPCR.

Multiple Sequence Alignment (MSA) was performed with the archaeal and bacterial *amoA* sequence obtained from Sanger sequencing, against the most closely related BLAST hits using the Clustal Omega program (70). The archaeal *amoA* sequence was aligned against the *amoA* sequence of Nitrososphaera viennensis, Nitrososphaera evergladensis and Nitrososphaera gargensis, and 2 pairs of Nitrososphaera *amoA* primers were designed from the consensus *amoA* sequence. The bacterial *amoA* sequence was aligned against the query *amoA* sequences of N. oligotropha and Nitrosomonas ureae, and 2 pairs of Nitrosomonas *amoA* primers were designed from the consensus *amoA* sequence. The primers were designed according to the guidelines specified for ddPCR provided by Bio-Rad Laboratories, Inc, with the primer sites selecting for a maximum amplicon size of 200 bp, and with melting temperatures between 61°C and 63°C.

The specificity of the newly designed primers was checked *in silico* using PRIMER-BLAST (71), and no cross-reactions with non-oligotrophic Nitrosomonas or non-Nitrososphaera AOA were found. The 2 primer pairs were mixed in equimolar concentrations for PCR applications.

Comparing the newly designed primer pairs to the published primers (Fig. S7 and S8), the new primers produced positive droplet events that were better separated from the negative droplet events, compared to those produced by the published primers.

### Experiment to validate washout of AOB with addition of DMTU and pyruvate into culture.

After the *amoA* concentrations of the AOA and AOB culture stocks were obtained using ddPCR, aliquots of the stock cultures were diluted and combined for a mixed culture containing 10^3^ to 10^4^ genomic copies/mL. These AOA-AOB mixed cultures were separated into 3 groups: (i) With 100 μM DMTU and pyruvate; (ii) With 100 μM pyruvate; (iii) Without DMTU or pyruvate. The experiment was conducted in triplicate at ambient temperature (mean of 23°C) with a culture volume of 100 mL in 250-mL Erlenmeyer flasks, which were shaken at 150 rpm on an orbital shaker covered with black paper. The cultures were supplied with 4 mM NH_4_Cl and ammonia consumption was monitored with Ammonia (Nitrogen) Test Strips, 0-6 mg/L (HACH). Upon depletion of the supplied ammonia, biomass from 60 mL of culture was collected with the Sterivex-GP Pressure Filter Unit (Merck Millipore) and stored at −20°C, while 10 mL was subcultured into a total volume of 100 mL containing 4 mM NH_4_Cl. The experiment was terminated after 3 subculture events. AOA and AOB cells were quantified by ddPCR based on genomic copies per milliliter of archaeal or bacterial *amoA*.

### Nucleic acid extraction from enrichment culture.

Culture biomass was collected through filtration of 50 mL of culture through Sterivex-GP Pressure Filter Units (Merck Millipore), using a 50 mL sterile disposable syringe. The filter units were further dewatered by passing 50 mL of air through the filters using the same syringe. Extraction of nucleic acid was done on fresh or frozen filter units, using the DNeasy PowerWater Sterivax Kit (Qiagen) according to the manufacturer’s instructions.

### Cycles for qPCR and ddPCR.

We performed qPCR with the PowerUp Sybr green MasterMix (Applied Biosystems) using 200 nM primers and 5 μL of template in a 25-μL reaction volume. The reaction was performed in a StepOnePlus thermocycler (Applied Biosystems) with the following cycle conditions: 50°C for 2 min and 95°C for 5 min, followed by 40 cycles of 95°C for 30 s and 60°C for 1 min. Plasmids used as standards for absolute quantification containing Nitrososphaera viennensis *amoA* and N. oligotropha *amoA* were synthesized (Integrated DNA Technologies) for the detection of AOA and AOB, respectively.

The ddPCR reaction was performed using the QX200 ddPCR EvaGreen SuperMix (Bio-Rad) and 250 nM forward primer (100 nM for AOB), 100 nM reverse primer, and 5 μL of template in a 25-μL reaction volume. The droplet generation, PCR and droplet reading was performing using the QX200 AutoDG Droplet Digital PCR System (Bio-Rad) with the following cycle conditions: 95°C for 5 min, 40 cycles of 95°C for 30 s and 60°C for 1 min, 4°C for 5 min, 90°C for 5 min, and finally hold at 4°C (all steps with a ramp rate of 2°C s^−1^). The primers used in the experiments are listed in Table 2. Concentrations of the plasmid standards and DNA samples were measured with a Qubit 2.0 fluorometer, using the dsDNA HS (high sensitivity) assay kit (Invitrogen).

The qPCR and ddPCR reactions using Arch_amoAF/Arch_amoAR primers (72) were performed in a 25-μL reaction volume containing 12.5 μL of master mix (PowerUp Sybr green or EvaGreen SuperMix), 400 nM primers and 5 μL of template. The qPCR was performed with the cycle conditions: 50°C for 2 min, 95°C for 5 min, 40 cycles of 95°C for 30 s, 56°C for 1 min and 72°C for 1 min. The ddPCR reaction was performed with the following cycle conditions: 95°C for 5 min, 40 cycles of 95°C for 30 s, 56°C for 1 min and 72°C for 1 min, 4°C for 5 min, 90°C for 5 min, and finally hold at 4°C (All steps with a ramp rate of 2°C.s^−1^).

The qPCR and ddPCR reactions using amoA332F/amoA822R primers (73) involved a 25 μL reaction volume containing 12.5 μL of master mix (PowerUp Sybr green or EvaGreen SuperMix), 1 μM primers and 5 μL of template. The qPCR was performed with the following cycle conditions: 50°C for 2 min, 95°C for 5 min, 40 cycles of 95°C for 30 s, 56°C for 1 min and 72°C for 1 min. The ddPCR reaction was performed with the following cycle conditions: 95°C for 5 min, 40 cycles of 95°C for 30 s, 56°C for 1 min and 72°C for 1 min, 4°C for 5 min, 90°C for 5 min, and finally hold at 4°C (all steps with a ramp rate of 2°C.s^−1^).

### Fixation of AOA and AOB enrichment cultures.

Forty milliliters of the nitrifier cultures were collected in 50-mL centrifuge tubes and centrifuged at 16,000 × *g* for 10 min. The cell pellets were resuspended in 40 mL of 1x phosphate-buffered saline (PBS) buffer and centrifuged again at 16,000 × *g*. The washed pellets were resuspended in 1 mL of ice-cold 2% formaldehyde in 1x PBS buffer, and then incubated at 4°C for 3 h. After incubation, the fixed samples were washed with ice-cold 1x PBS buffer, followed by resuspension in 1 mL of an ice-cold solution containing one part 1x PBS and one part 96% ethanol before storage at −20°C.

### FISH of AOA and AOB.

Before performing *in situ* hybridization, the fixed samples stored in −20°C were vortexed briefly and 10 μL of suspension was applied onto sample wells of the Teflon coated microscopy slide with Adcell (Thermo Fischer) and dried in a 46°C oven for approximately 15 min. Afterwards, the slides were dipped sequentially into 50%, 80%, and 96% ethanol for 3 min each, followed by drying the slides in a 46°C oven until the samples were completely dehydrated.

The probes used for labeling AOA and AOB for confocal laser scanning microscopy (CLSM) are listed in Table 3; they were diluted to a 30 ng/μL working solution and kept on ice before performing *in situ* hybridization. Two microliter of each probe was added into 20 μL of Hybridization Buffer with 35% formamide (Per mL: 180 μL 5 M NaCl, 20 μL 1 M Tris-HCl, 350 μL formamide, 1 μL 10% SDS, 449 μL ddH_2_O) and then applied to each sample circle on the slide. The slide was then placed in a 50-mL centrifuge tube atop a paper tower soaked with the remaining volume of Hybridization Buffer; next the tube was capped tightly and placed horizontally on a rack placed in a 46°C oven and incubated for 2 h. After incubation, the slide was washed with Washing Buffer (Per 50 mL: 0.7 mL 5 M NaCl, 1 mL 1 M Tris-HCl, 0.5 mL 0.5 M EDTA, 47.8 mL ddH_2_O) prewarmed to 48°C, transferred into the remaining Washing Buffer, and incubated in a 48°C water bath for 10 min. The slides were quickly dipped in ice-cold ddH_2_O after incubation, then air dried under the flow of nitrogen gas to quickly dry the samples. One small drop of Prolong Diamond Antifade mountant with DAPI (Invitrogen) was applied per 4 circles on each slide, and then a microscopy cover slip was carefully placed onto the samples spreading the mountant evenly across each cell. The samples were left to cure for 24 h before microscopic observation.

**TABLE 3:** Oligonucleotide probes targeting 16S rRNA used in this study

Probe	Fluorophore^*a*^	Sequence (5′–3′)	Competitor sequence^*b*^ (5′–3′)	FA^*c*^ (%)	Target	Source
S-D-Arch-0915-a-A-20	Alexa Fluor 448	GTGCTCCCCCGCCAATTCCT		35	Archaea	74, 77
Nm_OL_703	Cy5	GCCATCGATGTTCTTCCATATCTC	GCCATCGGTGTTCCTCCATATCTC GCCATCGGTGTTCCTCCACATCTC	35	*N. oligotropha,* *N. ureae,* *N. aestuarii,* *N. marina* lineage (cluster 6a + 6b)	75, 78

a Probes were labeled at the 5’ and 3’ ends.

b Competitors were unlabeled oligonucleotides and added at the same molar concentrations as the labeled probes.

c Formamide, with the concentration in the hybridization buffer (vol/vol) indicated in parentheses.

### Confocal laser scanning microscopy.

The samples were observed with a Carl Zeiss LSM 780, utilizing the 100× objective with oil immersion, and the software ZEN 2.3 SP1 FP3 Black (Carl Zeiss), using an Airy Unit of 1 for all scans. Image acquisition was performed with as a z-stack at maximum scan speed, using a frame size of 512 × 512 pixels and an average of 2 with Line mode, Mean method, and 8 Bit depth, to obtain an image size of 85.0 μm × 85.0 μm.

Image processing of the CLSM images was done with the Imaris ver. 9.0.0 (Bitplane AG). In brief, the image was loaded into the “Surpass” function with “3D View” and “Smoothing” was performed with “Median Filter” on all channels with the filter size of 3 × 3 × 1, followed by “Background Subtraction” on all channels with the default “Filter Width”. “Volume” and “Ortho Slicer” was checked under “Scene,” with the “XY Plane” option was checked under “Slice Orientation,” the maximum thickness chosen for “Extended Section” and “Slice Position,” and the “Show Frame” option unchecked. The scale bar was adjusted accordingly before the image was exported as a TIFF image with the “Snapshot” function.
