# Supplementary material for: Selective Enrichment of Nitrososphaera viennensis-Like Ammonia-Oxidizing Archaea over Ammonia-Oxidizing Bacteria from Drinking Water Biofilms
Source: Microbiol Spectr. 2022 Nov 29;10(6):e01845-22. doi: 10.1128/spectrum.01845-22 (PMC9769795; doi:10.1128/spectrum.01845-22)
Supplement: Supplemental file 1 — Fig. S1 to S14. Download spectrum.01845-22-s0001.pdf, PDF file, 4.4 MB [file spectrum.01845-22-s0001.pdf]

Supplemental Material (Figs S1 – S14)

Selective Enrichment of *Nitrososphaera viennensis*-like Ammonia-Oxidizing Archaea over Ammonia-Oxidizing Bacteria from Drinking Water Biofilms

*Yissue Woo[a,b,c], Mercedes Cecilia Cruz[a#], and Stefan Wuertz[a,c]**

[a]Singapore Centre for Environmental Life Sciences Engineering, Nanyang Technological University, Singapore

[b]Singapore Centre for Environmental Life Sciences Engineering, Interdisciplinary Graduate Programme, Nanyang Technological University, Singapore

[c]School of Civil and Environmental Engineering, Nanyang Technological University, Singapore

* Address correspondence to: Stefan Wuertz, swuertz@ntu.edu.sg

[#] Present Address: (1) Instituto de Investigaciones para la Industria Química (INIQUI), Consejo Nacional de Investigaciones Científicas y Técnicas (CONICET) National Scientific and Technical Research Council of Argentina (CONICET), Godoy Cruz 2290, Piso 9 (C1425FQB), Ciudad Autónoma de Buenos Aires, República Argentina. (2) Department of Biological Sciences, Marquette University, 1428 W Clybourn Street, Milwaukee, Wisconsin 53233, US. Mail: mccruz@conicet.gov.ar

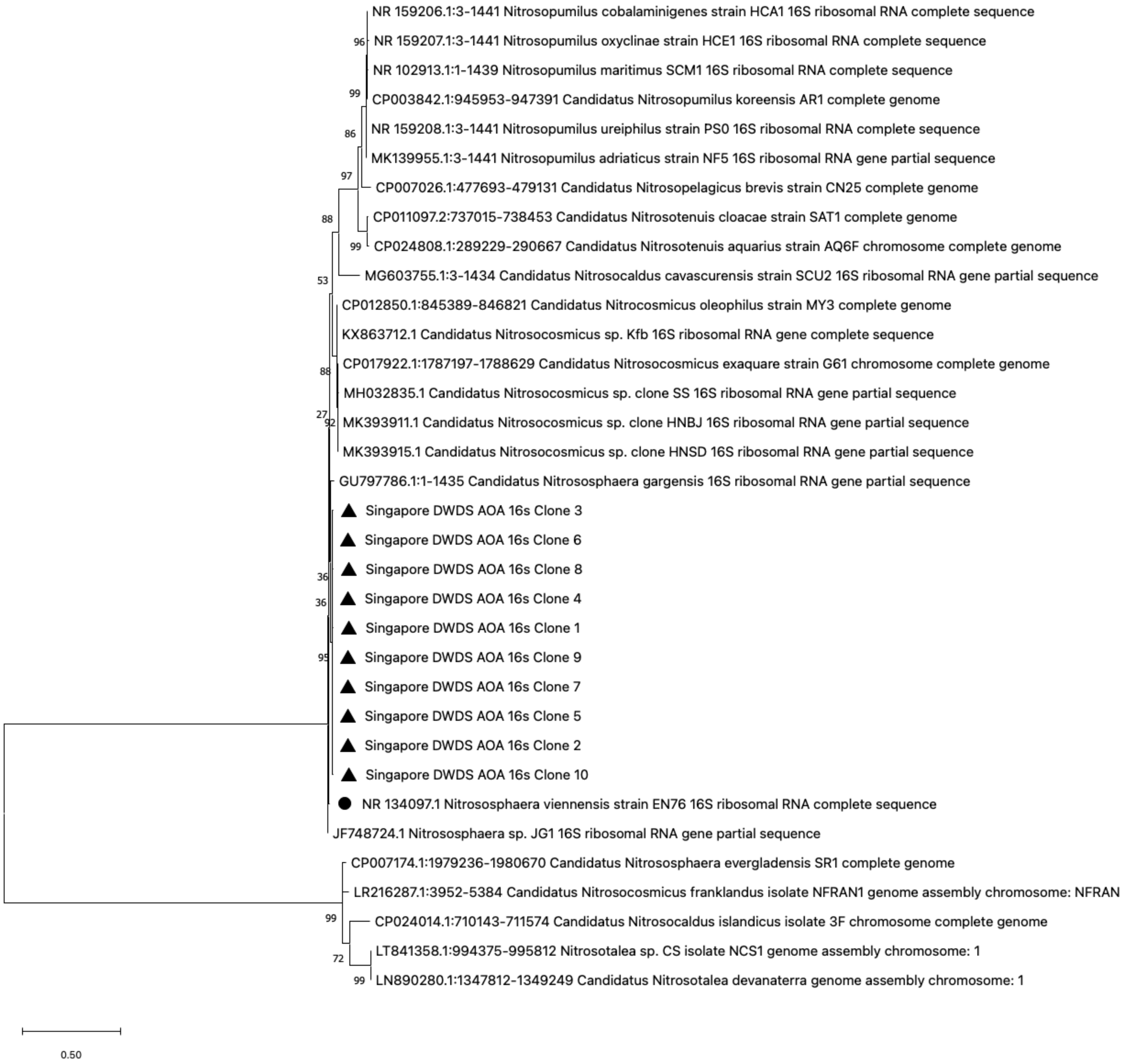
NR 159206.1:3-1441 Nitrosopumilus cobalaminigenes strain HCA1 16S ribosomal RNA complete sequence
NR 159207.1:3-1441 Nitrosopumilus oxyclinae strain HCE1 16S ribosomal RNA complete sequence
NR 102913.1:1-1439 Nitrosopumilus maritimus SCM1 16S ribosomal RNA complete sequence
CP003842.1:945953-947391 Candidatus Nitrosopumilus koreensis AR1 complete genome
NR 159208.1:3-1441 Nitrosopumilus ureiphilus strain PS0 16S ribosomal RNA complete sequence
MK139955.1:3-1441 Nitrosopumilus adriaticus strain NF5 16S ribosomal RNA gene partial sequence
CP007026.1:477693-479131 Candidatus Nitrosopelagicus brevis strain CN25 complete genome
CP011097.2:737015-738453 Candidatus Nitrosotenuis cloacae strain SAT1 complete genome
CP024808.1:289229-290667 Candidatus Nitrosotenuis aquarius strain AQ6F chromosome complete genome
MG603755.1:3-1434 Candidatus Nitrosocaldus cavascurensis strain SCU2 16S ribosomal RNA gene partial sequence
CP012850.1:845389-846821 Candidatus Nitrocosmicus oleophilus strain MY3 complete genome
KX863712.1 Candidatus Nitrosocosmicus sp. Kfb 16S ribosomal RNA gene complete sequence
CP017922.1:1787197-1788629 Candidatus Nitrosocosmicus exaquare strain G61 chromosome complete genome
MH032835.1 Candidatus Nitrosocosmicus sp. clone SS 16S ribosomal RNA gene partial sequence
MK393911.1 Candidatus Nitrosocosmicus sp. clone HNBJ 16S ribosomal RNA gene partial sequence
MK393915.1 Candidatus Nitrosocosmicus sp. clone HNSD 16S ribosomal RNA gene partial sequence
GU797786.1:1-1435 Candidatus Nitrososphaera gargensis 16S ribosomal RNA gene partial sequence
Singapore DWDS AOA 16s Clone 3
Singapore DWDS AOA 16s Clone 6
Singapore DWDS AOA 16s Clone 8
Singapore DWDS AOA 16s Clone 4
Singapore DWDS AOA 16s Clone 1
Singapore DWDS AOA 16s Clone 9
Singapore DWDS AOA 16s Clone 7
Singapore DWDS AOA 16s Clone 5
Singapore DWDS AOA 16s Clone 2
Singapore DWDS AOA 16s Clone 10
NR 134097.1 Nitrososphaera viennensis strain EN76 16S ribosomal RNA complete sequence
JF748724.1 Nitrososphaera sp. JG1 16S ribosomal RNA gene partial sequence
CP007174.1:1979236-1980670 Candidatus Nitrososphaera evergladensis SR1 complete genome
LR216287.1:3952-5384 Candidatus Nitrosocosmicus franklandus isolate NFRAN1 genome assembly chromosome: NFRAN
CP024014.1:710143-711574 Candidatus Nitrosocaldus islandicus isolate 3F chromosome complete genome
LT841358.1:994375-995812 Nitrosotalea sp. CS isolate NCS1 genome assembly chromosome: 1
LN890280.1:1347812-1349249 Candidatus Nitrosotalea devanaterra genome assembly chromosome: 1
96
99
86
97
88
99
53
88
27
92
36
36
95
99
72
99
0.50

Figure S1. Maximum likelihood phylogenetic tree based on archaeal 16S rRNA gene nucleotide sequences. The cloned archaeal 16S rRNA sequences are most related to the 16S rRNA gene sequence from *Nitrososphaera viennensis* strain EN76 (closed circle). The evolutionary history was inferred by using the Maximum Likelihood method and Tamura-Nei model (80). The unrooted tree with the highest log likelihood (-8752.35) is shown. Initial tree(s) for the heuristic search were obtained automatically by applying Neighbor-Join and BioNJ algorithms to a matrix of pairwise distances estimated using the Maximum Composite Likelihood (MCL) approach, and then selecting the topology with superior log likelihood value. A discrete Gamma distribution was used to model evolutionary rate differences among sites (5 categories (+G, parameter = 1.0804)). The tree is drawn to scale, with branch lengths measured in the number of substitutions per site. This analysis involved 34 nucleotide sequences. All positions with less than 90% site coverage were eliminated, i.e., fewer than 10% alignment gaps, missing data, and ambiguous bases were allowed at any position (partial deletion option). There was a total of 1397 positions in the final dataset. The number of bootstrap replications was 500. Evolutionary analyses were conducted in MEGA X (67, 81).

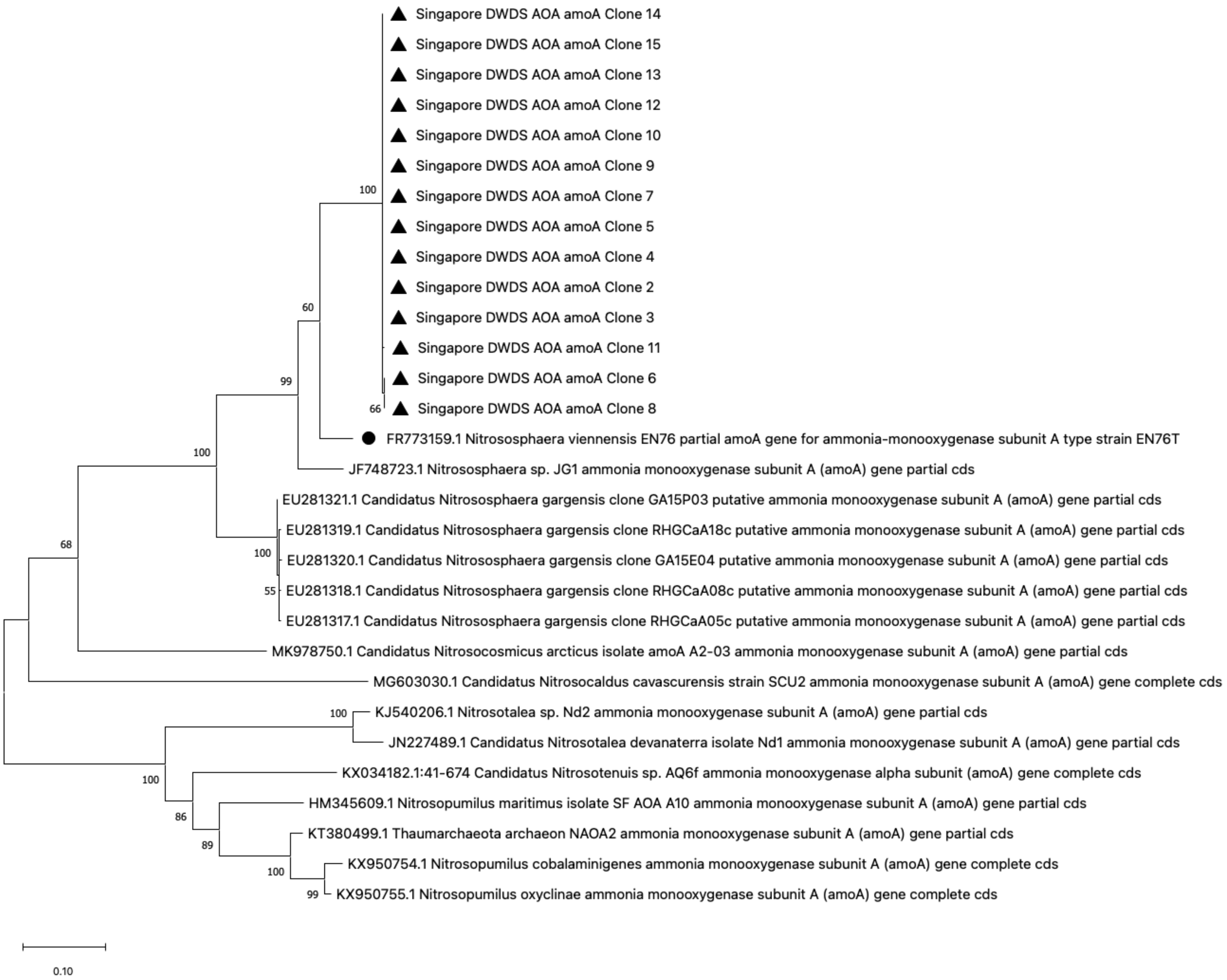
Singapore DWDS AOA amoA Clone 14
Singapore DWDS AOA amoA Clone 15
Singapore DWDS AOA amoA Clone 13
Singapore DWDS AOA amoA Clone 12
Singapore DWDS AOA amoA Clone 10
Singapore DWDS AOA amoA Clone 9
Singapore DWDS AOA amoA Clone 7
Singapore DWDS AOA amoA Clone 5
Singapore DWDS AOA amoA Clone 4
Singapore DWDS AOA amoA Clone 2
Singapore DWDS AOA amoA Clone 3
Singapore DWDS AOA amoA Clone 11
Singapore DWDS AOA amoA Clone 6
Singapore DWDS AOA amoA Clone 8
FR773159.1 Nitrososphaera viennensis EN76 partial amoA gene for ammonia-monooxygenase subunit A type strain EN76T
JF748723.1 Nitrososphaera sp. JG1 ammonia monooxygenase subunit A (amoA) gene partial cds
EU281321.1 Candidatus Nitrososphaera gargensis clone GA15P03 putative ammonia monooxygenase subunit A (amoA) gene partial cds
EU281319.1 Candidatus Nitrososphaera gargensis clone RHGCaA18c putative ammonia monooxygenase subunit A (amoA) gene partial cds
EU281320.1 Candidatus Nitrososphaera gargensis clone GA15E04 putative ammonia monooxygenase subunit A (amoA) gene partial cds
EU281318.1 Candidatus Nitrososphaera gargensis clone RHGCaA08c putative ammonia monooxygenase subunit A (amoA) gene partial cds
EU281317.1 Candidatus Nitrososphaera gargensis clone RHGCaA05c putative ammonia monooxygenase subunit A (amoA) gene partial cds
MK978750.1 Candidatus Nitrosocosmicus arcticus isolate amoA A2-03 ammonia monooxygenase subunit A (amoA) gene partial cds
MG603030.1 Candidatus Nitrosocaldus cavascurensis strain SCU2 ammonia monooxygenase subunit A (amoA) gene complete cds
KJ540206.1 Nitrosotalea sp. Nd2 ammonia monooxygenase subunit A (amoA) gene partial cds
JN227489.1 Candidatus Nitrosotalea devanaterra isolate Nd1 ammonia monooxygenase subunit A (amoA) gene partial cds
KX034182.1:41-674 Candidatus Nitrosotenuis sp. AQ6f ammonia monooxygenase alpha subunit (amoA) gene complete cds
HM345609.1 Nitrosopumilus maritimus isolate SF AOA A10 ammonia monooxygenase subunit A (amoA) gene partial cds
KT380499.1 Thaumarchaeota archaeon NAOA2 ammonia monooxygenase subunit A (amoA) gene partial cds
KX950754.1 Nitrosopumilus cobalaminigenes ammonia monooxygenase subunit A (amoA) gene complete cds
KX950755.1 Nitrosopumilus oxyclinae ammonia monooxygenase subunit A (amoA) gene complete cds
100
60
99
66
100
100
55
68
100
100
86
89
100
99
0.10

Figure S2. Maximum likelihood phylogenetic tree based on archaeal *amoA* gene nucleotide sequences. The cloned archaeal *amoA* sequences (closed triangles) are most closely related to the *amoA* sequence from *Nitrososphaera viennensis* strain EN76 (closed circle). The evolutionary history was inferred by using the Maximum Likelihood method and General Time Reversible model (80). The unrooted tree with the highest log likelihood (-4008.34) is shown. The percentage of trees in which the associated taxa clustered together is shown next to the branches. Initial tree(s) for the heuristic search were obtained automatically by applying Neighbor-Join and BioNJ algorithms to a matrix of pairwise distances estimated using the Maximum Composite Likelihood (MCL) approach, and then selecting the topology with superior log likelihood value. A discrete Gamma distribution was used to model evolutionary rate differences among sites (5 categories (+G, parameter = 1.0704)). The rate variation model allowed for some sites to be evolutionarily invariable ([+I], 37.69% sites). The tree is drawn to scale, with branch lengths measured in the number of substitutions per site. This analysis involved 30 nucleotide sequences. Codon positions included were 1st+2nd+3rd+Noncoding. All positions with less than 90% site coverage were eliminated, i.e., fewer than 10% alignment gaps, missing data, and ambiguous bases were allowed at any position (partial deletion option). There was a total of 600 positions in the final dataset. The number of bootstrap replications was 500. Evolutionary analyses were conducted in MEGA X (67, 81).

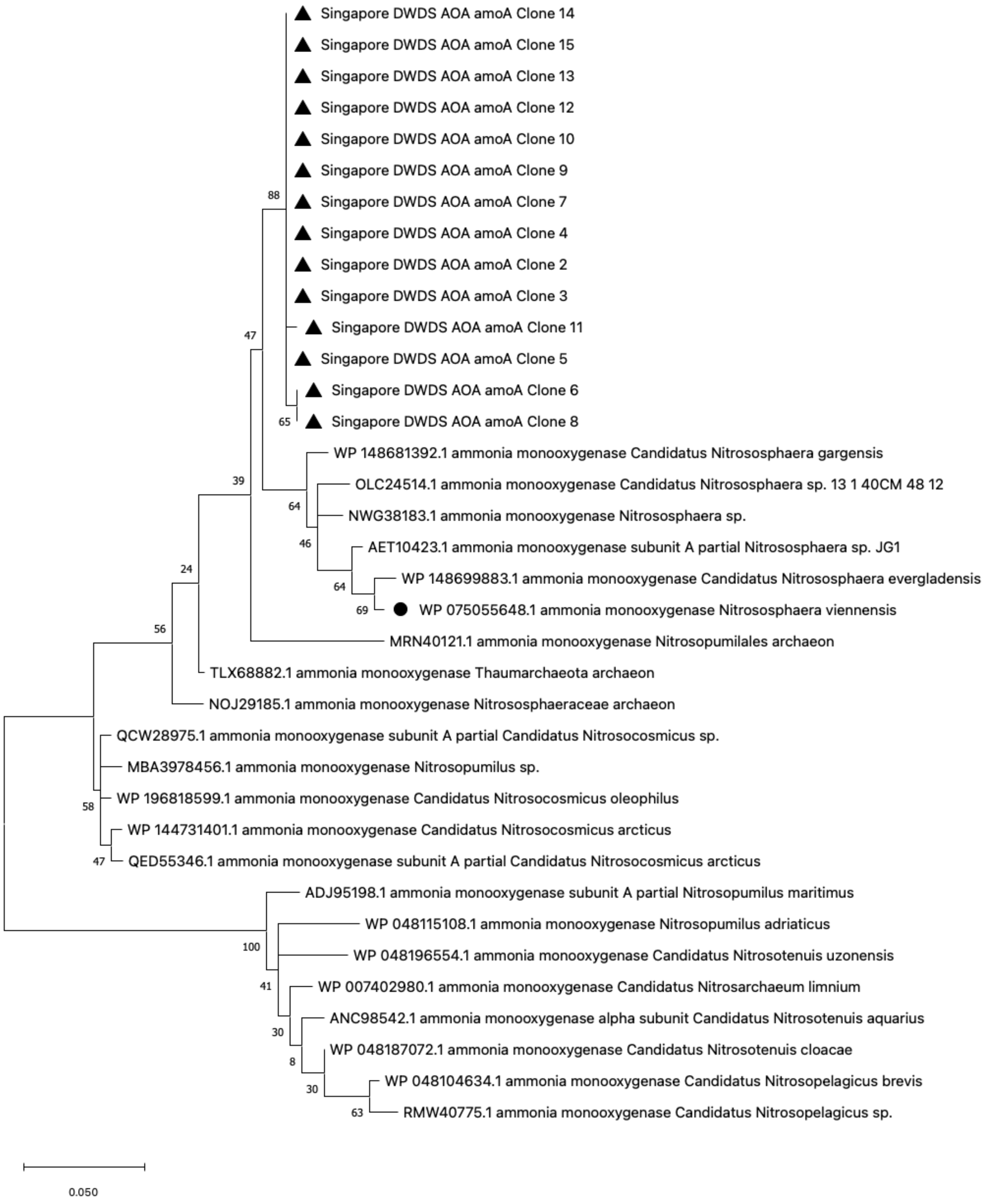

Singapore DWDS AOA amoA Clone 14
Singapore DWDS AOA amoA Clone 15
Singapore DWDS AOA amoA Clone 13
Singapore DWDS AOA amoA Clone 12
Singapore DWDS AOA amoA Clone 10
Singapore DWDS AOA amoA Clone 9
Singapore DWDS AOA amoA Clone 7
Singapore DWDS AOA amoA Clone 4
Singapore DWDS AOA amoA Clone 2
Singapore DWDS AOA amoA Clone 3
Singapore DWDS AOA amoA Clone 11
Singapore DWDS AOA amoA Clone 5
Singapore DWDS AOA amoA Clone 6
Singapore DWDS AOA amoA Clone 8
WP 148681392.1 ammonia monooxygenase Candidatus Nitrososphaera gargensis
OLC24514.1 ammonia monooxygenase Candidatus Nitrososphaera sp. 13 1 40CM 48 12
NWG38183.1 ammonia monooxygenase Nitrososphaera sp.
AET10423.1 ammonia monooxygenase subunit A partial Nitrososphaera sp. JG1
WP 148699883.1 ammonia monooxygenase Candidatus Nitrososphaera evergladensis
WP 075055648.1 ammonia monooxygenase Nitrososphaera viennensis
MRN40121.1 ammonia monooxygenase Nitrosopumilales archaeon
TLX68882.1 ammonia monooxygenase Thaumarchaeota archaeon
NOJ29185.1 ammonia monooxygenase Nitrososphaeraceae archaeon
QCW28975.1 ammonia monooxygenase subunit A partial Candidatus Nitrosocosmicus sp.
MBA3978456.1 ammonia monooxygenase Nitrosopumilus sp.
WP 196818599.1 ammonia monooxygenase Candidatus Nitrosocosmicus oleophilus
WP 144731401.1 ammonia monooxygenase Candidatus Nitrosocosmicus arcticus
QED55346.1 ammonia monooxygenase subunit A partial Candidatus Nitrosocosmicus arcticus
ADJ95198.1 ammonia monooxygenase subunit A partial Nitrosopumilus maritimus
WP 048115108.1 ammonia monooxygenase Nitrosopumilus adriaticus
WP 048196554.1 ammonia monooxygenase Candidatus Nitrosotenuis uzonensis
WP 007402980.1 ammonia monooxygenase Candidatus Nitrosarchaeum limnium
ANC98542.1 ammonia monooxygenase alpha subunit Candidatus Nitrosotenuis aquarius
WP 048187072.1 ammonia monooxygenase Candidatus Nitrosotenuis cloacae
WP 048104634.1 ammonia monooxygenase Candidatus Nitrosopelagicus brevis
RMW40775.1 ammonia monooxygenase Candidatus Nitrosopelagicus sp.
88
65
47
39
64
46
64
69
24
56
58
47
100
41
30
8
30
63
0.050

Figure S3. Maximum likelihood phylogenetic tree based on translated peptide sequences of cloned archaeal *amoA* nucleotide sequences. The translated peptide sequences of the cloned archaeal *amoA* are closely related to the *amoA* peptide sequences from the AOA genera *Nitrososphaera*, including the species *Nitrososphaera viennensis* (closed circle). The cloned sequences are highlighted by closed triangles. The evolutionary history were inferred using the Maximum Likelihood method (80) and Le_Gascuel_2008 model (82). The unrooted tree with the highest log likelihood (-1321.91) is shown. Initial tree(s) for the heuristic search were obtained automatically by applying Neighbor-Join and BioNJ algorithms to a matrix of pairwise distances estimated using a JTT model, and then selecting the topology with superior log likelihood value. A discrete Gamma distribution was used to model evolutionary rate differences among sites (5 categories (+G, parameter = 0.2277)). The tree is drawn to scale, with branch lengths measured in the number of substitutions per site. This analysis involved 36 amino acid sequences. All positions with less than 90% site coverage were eliminated, i.e., fewer than 10% alignment gaps, missing data, and ambiguous bases were allowed at any position (partial deletion option). The final dataset had a total of 210 positions. The number of bootstrap replications was 500. Evolutionary analyses were conducted in MEGA X (67, 81).

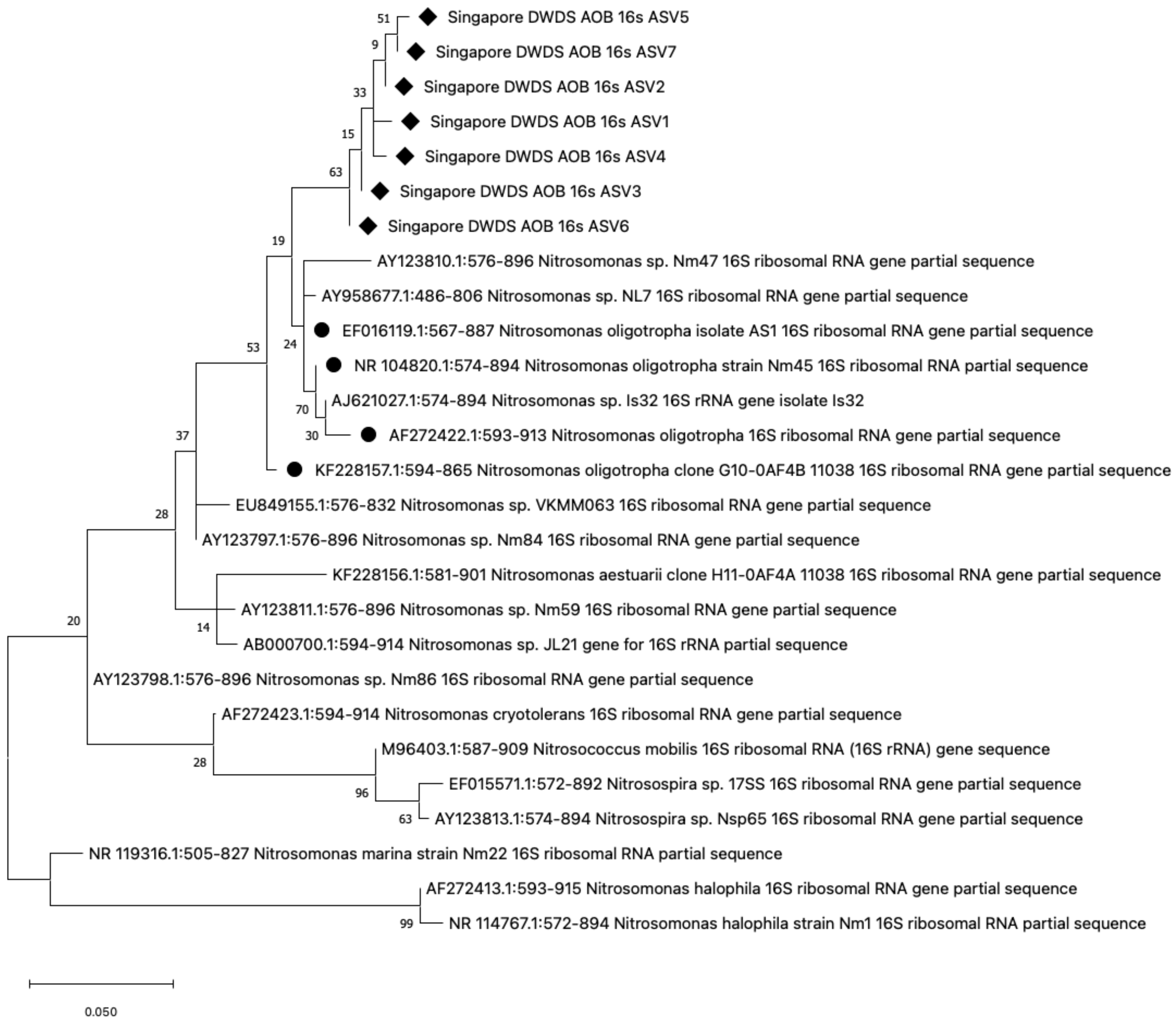
Singapore DWDS AOB 16s ASV5
Singapore DWDS AOB 16s ASV7
Singapore DWDS AOB 16s ASV2
Singapore DWDS AOB 16s ASV1
Singapore DWDS AOB 16s ASV4
Singapore DWDS AOB 16s ASV3
Singapore DWDS AOB 16s ASV6
AY123810.1:576-896 Nitrosomonas sp. Nm47 16S ribosomal RNA gene partial sequence
AY958677.1:486-806 Nitrosomonas sp. NL7 16S ribosomal RNA gene partial sequence
EF016119.1:567-887 Nitrosomonas oligotropha isolate AS1 16S ribosomal RNA gene partial sequence
NR 104820.1:574-894 Nitrosomonas oligotropha strain Nm45 16S ribosomal RNA partial sequence
AJ621027.1:574-894 Nitrosomonas sp. Is32 16S rRNA gene isolate Is32
AF272422.1:593-913 Nitrosomonas oligotropha 16S ribosomal RNA gene partial sequence
KF228157.1:594-865 Nitrosomonas oligotropha clone G10-0AF4B 11038 16S ribosomal RNA gene partial sequence
EU849155.1:576-832 Nitrosomonas sp. VKMM063 16S ribosomal RNA gene partial sequence
AY123797.1:576-896 Nitrosomonas sp. Nm84 16S ribosomal RNA gene partial sequence
KF228156.1:581-901 Nitrosomonas aestuarii clone H11-0AF4A 11038 16S ribosomal RNA gene partial sequence
AY123811.1:576-896 Nitrosomonas sp. Nm59 16S ribosomal RNA gene partial sequence
AB000700.1:594-914 Nitrosomonas sp. JL21 gene for 16S rRNA partial sequence
AY123798.1:576-896 Nitrosomonas sp. Nm86 16S ribosomal RNA gene partial sequence
AF272423.1:594-914 Nitrosomonas cryotolerans 16S ribosomal RNA gene partial sequence
M96403.1:587-909 Nitrosococcus mobilis 16S ribosomal RNA (16S rRNA) gene sequence
EF015571.1:572-892 Nitrosospira sp. 17SS 16S ribosomal RNA gene partial sequence
AY123813.1:574-894 Nitrosospira sp. Nsp65 16S ribosomal RNA gene partial sequence
NR 119316.1:505-827 Nitrosomonas marina strain Nm22 16S ribosomal RNA partial sequence
AF272413.1:593-915 Nitrosomonas halophila 16S ribosomal RNA gene partial sequence
NR 114767.1:572-894 Nitrosomonas halophila strain Nm1 16S ribosomal RNA partial sequence
51
9
33
15
63
19
24
53
70
30
37
28
14
20
28
96
63
99
0.050

Figure S4. Maximum likelihood phylogenetic tree based on 16S amplicon sequence variant (ASV) nucleotide sequences, which are closely related to the 16S sequences from the *Nitrosomonas oligotropha* group (closed circles) of AOB. The ASV sequences are highlighted by the filled diamonds. The evolutionary history was inferred by using the Maximum Likelihood method and Kimura 2-parameter model (80). The unrooted tree with the highest log likelihood (-883.41) is shown. The percentage of trees in which the associated taxa clustered together is shown next to the branches. Initial tree(s) for the heuristic search were obtained automatically by applying Neighbor-Join and BioNJ algorithms to a matrix of pairwise distances estimated using the Maximum Composite Likelihood (MCL) approach, and then selecting the topology with superior log likelihood value. A discrete Gamma distribution was used to model evolutionary rate differences among sites (5 categories (+G, parameter = 0.7320)). The rate variation model allowed for some sites to be evolutionarily invariable ([+I], 43.93% sites). The tree is drawn to scale, with branch lengths measured in the number of substitutions per site. This analysis involved 27 nucleotide sequences. All positions with less than 90% site coverage were eliminated, i.e., fewer than 10% alignment gaps, missing data, and ambiguous bases were allowed at any position (partial deletion option). There were a total of 321 positions in the final dataset. The number of bootstrap replications was 500. Evolutionary analyses were conducted in MEGA X (67, 81).

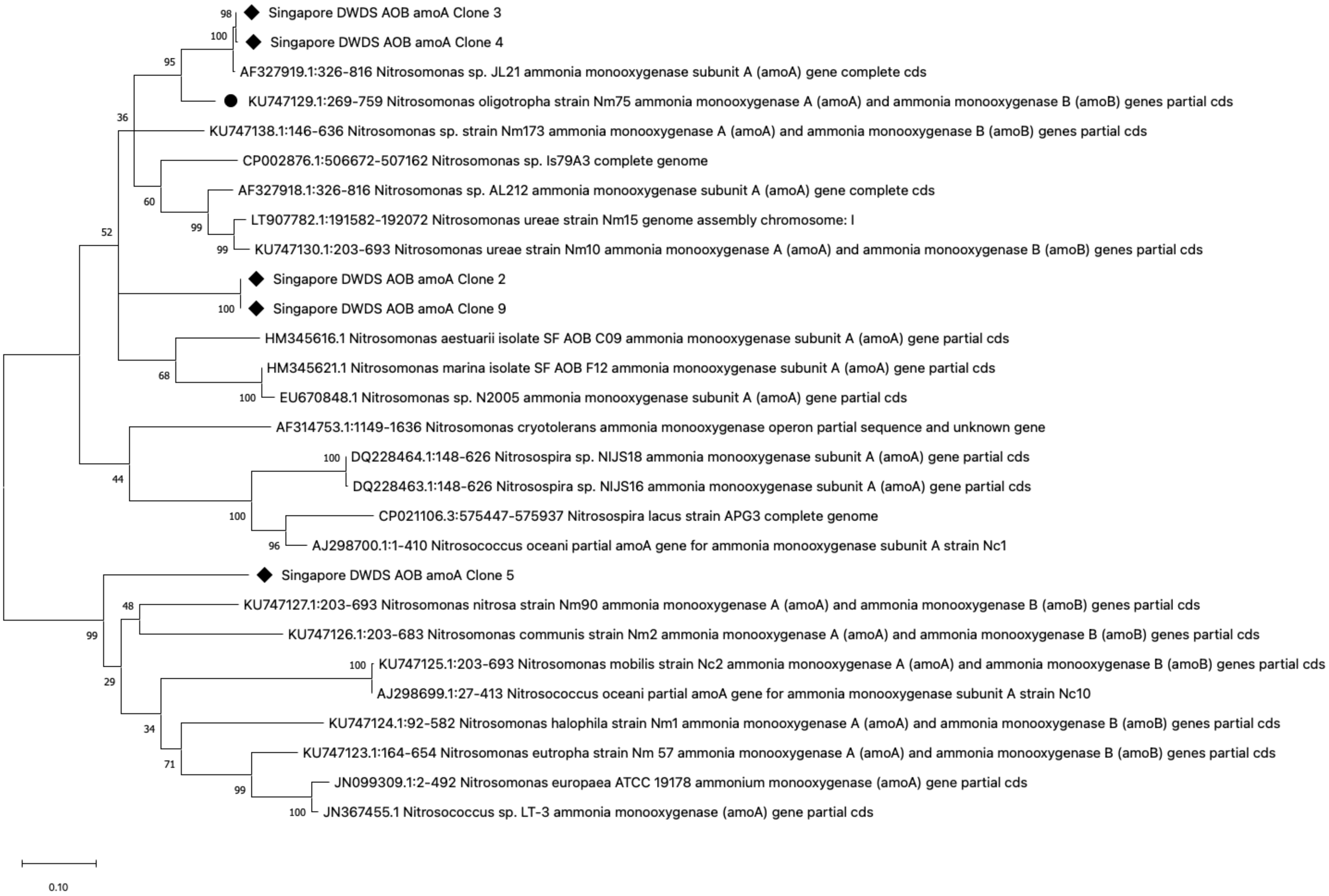
Singapore DWDS AOB amoA Clone 3
Singapore DWDS AOB amoA Clone 4
AF327919.1:326-816 Nitrosomonas sp. JL21 ammonia monooxygenase subunit A (amoA) gene complete cds
KU747129.1:269-759 Nitrosomonas oligotropha strain Nm75 ammonia monooxygenase A (amoA) and ammonia monooxygenase B (amoB) genes partial cds
KU747138.1:146-636 Nitrosomonas sp. strain Nm173 ammonia monooxygenase A (amoA) and ammonia monooxygenase B (amoB) genes partial cds
CP002876.1:506672-507162 Nitrosomonas sp. Is79A3 complete genome
AF327918.1:326-816 Nitrosomonas sp. AL212 ammonia monooxygenase subunit A (amoA) gene complete cds
LT907782.1:191582-192072 Nitrosomonas ureae strain Nm15 genome assembly chromosome: I
KU747130.1:203-693 Nitrosomonas ureae strain Nm10 ammonia monooxygenase A (amoA) and ammonia monooxygenase B (amoB) genes partial cds
Singapore DWDS AOB amoA Clone 2
Singapore DWDS AOB amoA Clone 9
HM345616.1 Nitrosomonas aestuarii isolate SF AOB C09 ammonia monooxygenase subunit A (amoA) gene partial cds
HM345621.1 Nitrosomonas marina isolate SF AOB F12 ammonia monooxygenase subunit A (amoA) gene partial cds
EU670848.1 Nitrosomonas sp. N2005 ammonia monooxygenase subunit A (amoA) gene partial cds
AF314753.1:1149-1636 Nitrosomonas cryotolerans ammonia monooxygenase operon partial sequence and unknown gene
DQ228464.1:148-626 Nitrosospira sp. NIJS18 ammonia monooxygenase subunit A (amoA) gene partial cds
DQ228463.1:148-626 Nitrosospira sp. NIJS16 ammonia monooxygenase subunit A (amoA) gene partial cds
CP021106.3:575447-575937 Nitrosospira lacus strain APG3 complete genome
AJ298700.1:1-410 Nitrosococcus oceani partial amoA gene for ammonia monooxygenase subunit A strain Nc1
Singapore DWDS AOB amoA Clone 5
KU747127.1:203-693 Nitrosomonas nitrosa strain Nm90 ammonia monooxygenase A (amoA) and ammonia monooxygenase B (amoB) genes partial cds
KU747126.1:203-683 Nitrosomonas communis strain Nm2 ammonia monooxygenase A (amoA) and ammonia monooxygenase B (amoB) genes partial cds
KU747125.1:203-693 Nitrosomonas mobilis strain Nc2 ammonia monooxygenase A (amoA) and ammonia monooxygenase B (amoB) genes partial cds
AJ298699.1:27-413 Nitrosococcus oceani partial amoA gene for ammonia monooxygenase subunit A strain Nc10
KU747124.1:92-582 Nitrosomonas halophila strain Nm1 ammonia monooxygenase A (amoA) and ammonia monooxygenase B (amoB) genes partial cds
KU747123.1:164-654 Nitrosomonas eutropha strain Nm 57 ammonia monooxygenase A (amoA) and ammonia monooxygenase B (amoB) genes partial cds
JN099309.1:2-492 Nitrosomonas europaea ATCC 19178 ammonium monooxygenase (amoA) gene partial cds
JN367455.1 Nitrosococcus sp. LT-3 ammonia monooxygenase (amoA) gene partial cds
0.10

Figure S5. Maximum likelihood phylogenetic tree based on the cloned bacterial *amoA* nucleotide sequences, which are diversely related to *amoA* nucleotide sequences from the AOB genera *Nitrosomonas* and *Nitrosococcus*, including the species *Nitrosomonas oligotropha* (closed circle). The cloned sequences are highlighted by the filled diamonds. The evolutionary history was inferred by using the Maximum Likelihood method and Kimura 2-parameter model (80). The unrooted tree with the highest log likelihood (-4809.00) is shown. The percentage of trees in which the associated taxa clustered together is shown next to the branches. Initial tree(s) for the heuristic search were obtained automatically by applying Neighbor-Join and BioNJ algorithms to a matrix of pairwise distances estimated using the Maximum Composite Likelihood (MCL) approach, and then selecting the topology with superior log likelihood value. A discrete Gamma distribution was used to model evolutionary rate differences among sites (5 categories (+G, parameter = 1.0460)). The rate variation model allowed for some sites to be evolutionarily invariable ([+I], 25.57% sites). The tree is drawn to scale, with branch lengths measured in the number of substitutions per site. This analysis involved 28 nucleotide sequences. All positions with less than 90% site coverage were eliminated, i.e., fewer than 10% alignment gaps, missing data, and ambiguous bases were allowed at any position (partial deletion option). There were a total of 479 positions in the final dataset. The number of bootstrap replications was 500. Evolutionary analyses were conducted in MEGA X (67, 81).

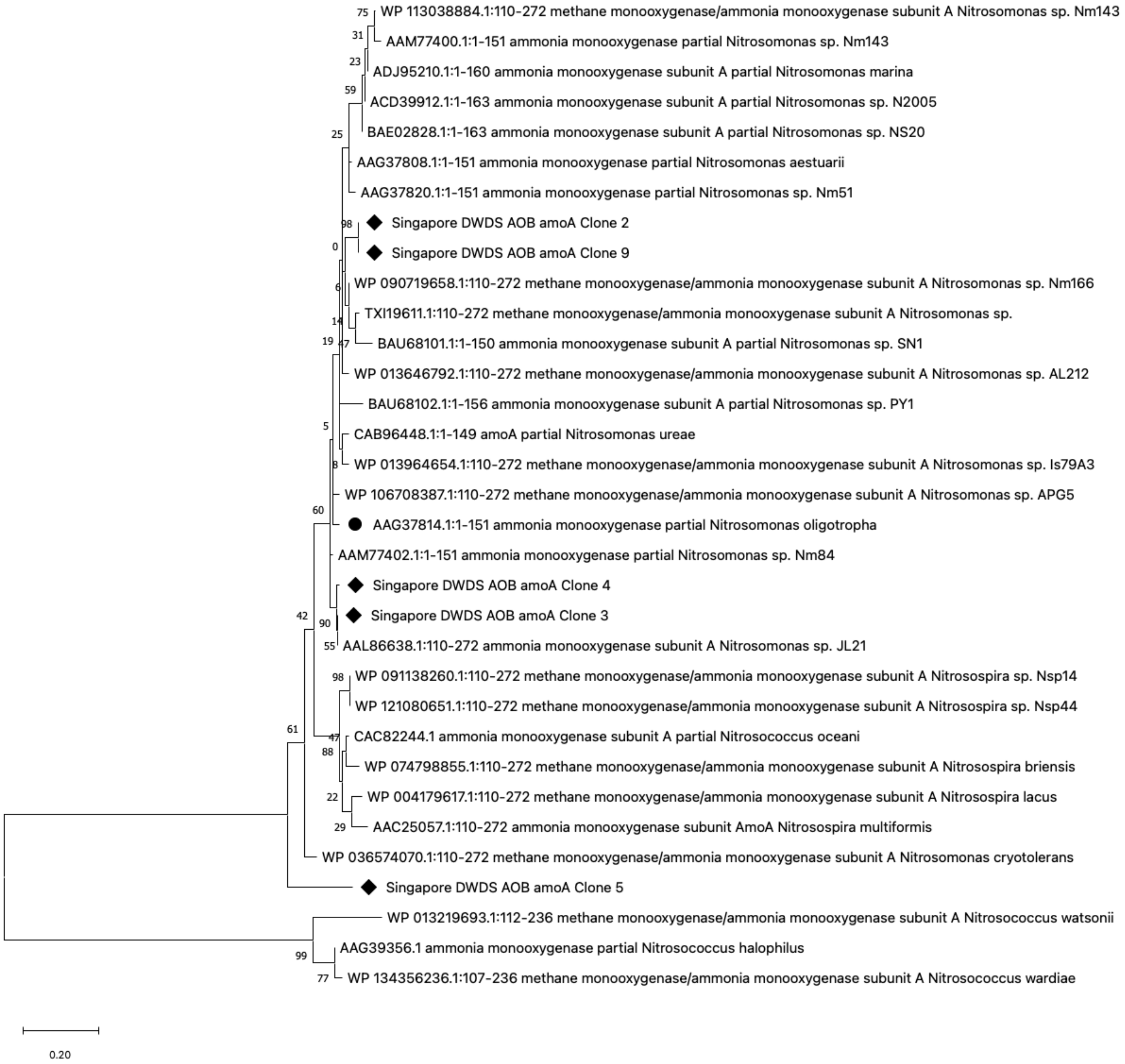
WP 113038884.1:110-272 methane monooxygenase/ammonia monooxygenase subunit A Nitrosomonas sp. Nm143
AAM77400.1:1-151 ammonia monooxygenase partial Nitrosomonas sp. Nm143
ADJ95210.1:1-160 ammonia monooxygenase subunit A partial Nitrosomonas marina
ACD39912.1:1-163 ammonia monooxygenase subunit A partial Nitrosomonas sp. N2005
BAE02828.1:1-163 ammonia monooxygenase subunit A partial Nitrosomonas sp. NS20
AAG37808.1:1-151 ammonia monooxygenase partial Nitrosomonas aestuarii
AAG37820.1:1-151 ammonia monooxygenase partial Nitrosomonas sp. Nm51
Singapore DWDS AOB amoA Clone 2
Singapore DWDS AOB amoA Clone 9
WP 090719658.1:110-272 methane monooxygenase/ammonia monooxygenase subunit A Nitrosomonas sp. Nm166
TXI19611.1:110-272 methane monooxygenase/ammonia monooxygenase subunit A Nitrosomonas sp.
BAU68101.1:1-150 ammonia monooxygenase subunit A partial Nitrosomonas sp. SN1
WP 013646792.1:110-272 methane monooxygenase/ammonia monooxygenase subunit A Nitrosomonas sp. AL212
BAU68102.1:1-156 ammonia monooxygenase subunit A partial Nitrosomonas sp. PY1
CAB96448.1:1-149 amoA partial Nitrosomonas ureae
WP 013964654.1:110-272 methane monooxygenase/ammonia monooxygenase subunit A Nitrosomonas sp. Is79A3
WP 106708387.1:110-272 methane monooxygenase/ammonia monooxygenase subunit A Nitrosomonas sp. APG5
AAG37814.1:1-151 ammonia monooxygenase partial Nitrosomonas oligotropha
AAM77402.1:1-151 ammonia monooxygenase partial Nitrosomonas sp. Nm84
Singapore DWDS AOB amoA Clone 4
Singapore DWDS AOB amoA Clone 3
AAL86638.1:110-272 ammonia monooxygenase subunit A Nitrosomonas sp. JL21
WP 091138260.1:110-272 methane monooxygenase/ammonia monooxygenase subunit A Nitrosospira sp. Nsp14
WP 121080651.1:110-272 methane monooxygenase/ammonia monooxygenase subunit A Nitrosospira sp. Nsp44
CAC82244.1 ammonia monooxygenase subunit A partial Nitrosococcus oceani
WP 074798855.1:110-272 methane monooxygenase/ammonia monooxygenase subunit A Nitrosospira briensis
WP 004179617.1:110-272 methane monooxygenase/ammonia monooxygenase subunit A Nitrosospira lacus
AAC25057.1:110-272 ammonia monooxygenase subunit AmoA Nitrosospira multiformis
WP 036574070.1:110-272 methane monooxygenase/ammonia monooxygenase subunit A Nitrosomonas cryotolerans
Singapore DWDS AOB amoA Clone 5
WP 013219693.1:112-236 methane monooxygenase/ammonia monooxygenase subunit A Nitrosococcus watsonii
AAG39356.1 ammonia monooxygenase partial Nitrosococcus halophilus
WP 134356236.1:107-236 methane monooxygenase/ammonia monooxygenase subunit A Nitrosococcus wardiae
0.20

Figure S6. Maximum likelihood phylogenetic tree based on translated peptide sequences of cloned bacterial amoA nucleotide sequences. The translated peptide sequences of the cloned bacterial amoA are closely related to the *amoA* peptide sequences from the AOB genera *Nitrosomonas*, including *Nitrosomonas oligotropha* (closed circle). The cloned sequences are highlighted by closed diamonds. One of the cloned sequences is diversely related to the *amoA* peptide sequences from the AOB genera *Nitrosomonas*, *Nitrosospira* and *Nitrosococcus*. The evolutionary history was inferred using the Maximum Likelihood method (80) and Le_Gascuel_2008 model (82). The unrooted tree with the highest log likelihood (-1454.32) is shown. Initial tree(s) for the heuristic search were obtained automatically by applying Neighbor-Join and BioNJ algorithms to a matrix of pairwise distances estimated using a JTT model, and then selecting the topology with superior log likelihood value. A discrete Gamma distribution was used to model evolutionary rate differences among sites (5 categories (+G, parameter = 0.7441)). The tree is drawn to scale, with branch lengths measured in the number of substitutions per site. This analysis involved 33 amino acid sequences. All positions with less than 90% site coverage were eliminated, i.e., fewer than 10% alignment gaps, missing data, and ambiguous bases were allowed at any position (partial deletion option). There was a total of 144 positions in the final dataset. The number of bootstrap replications was 500. Evolutionary analyses were conducted in MEGA X (67, 81).

A B

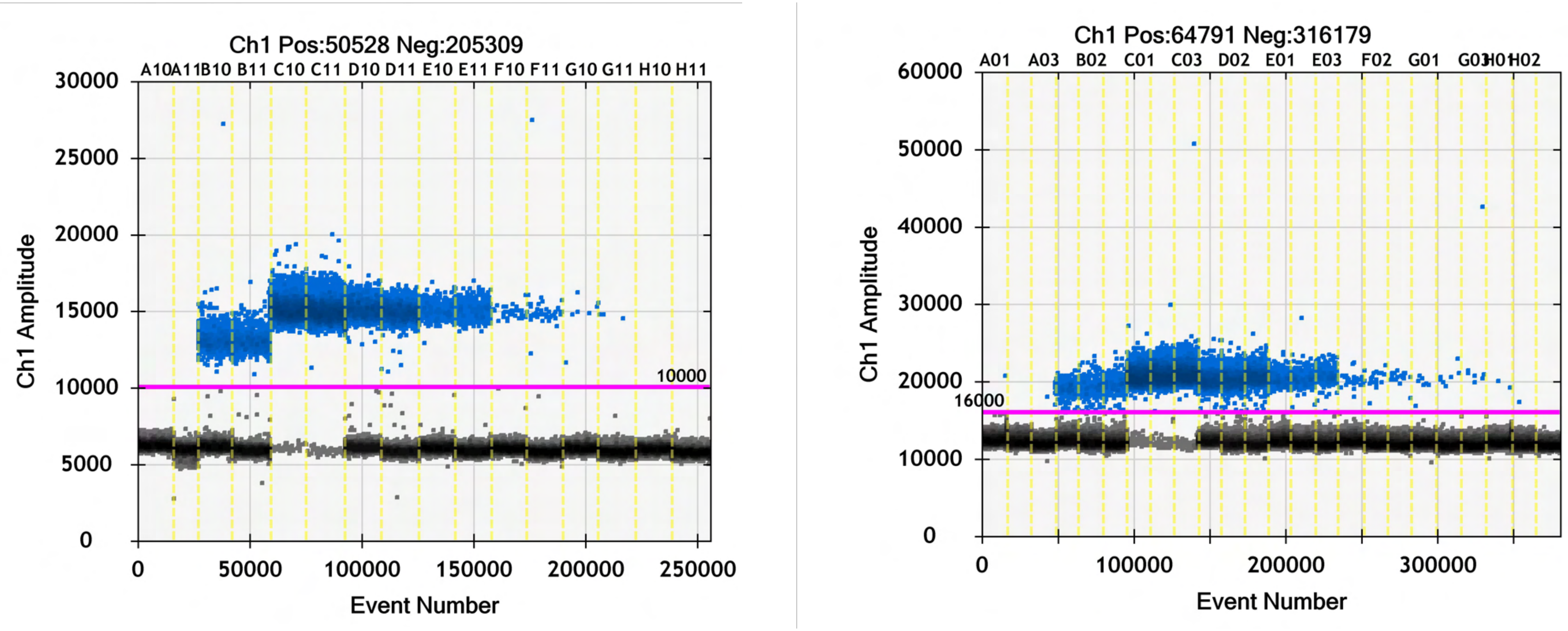

Figure S7. Comparison of ddPCR reactions using NSS_amoAF/R and Arch_amoAF/R primers. 1D plots from the archaeal *amoA* ddPCR droplet reads showed good signal separation for the positive droplets from the negative droplets of the reactions using the designed primers (A), but the signal separation between the positive and negative droplets of the reactions using the published primers (71) was poorer (B).

A

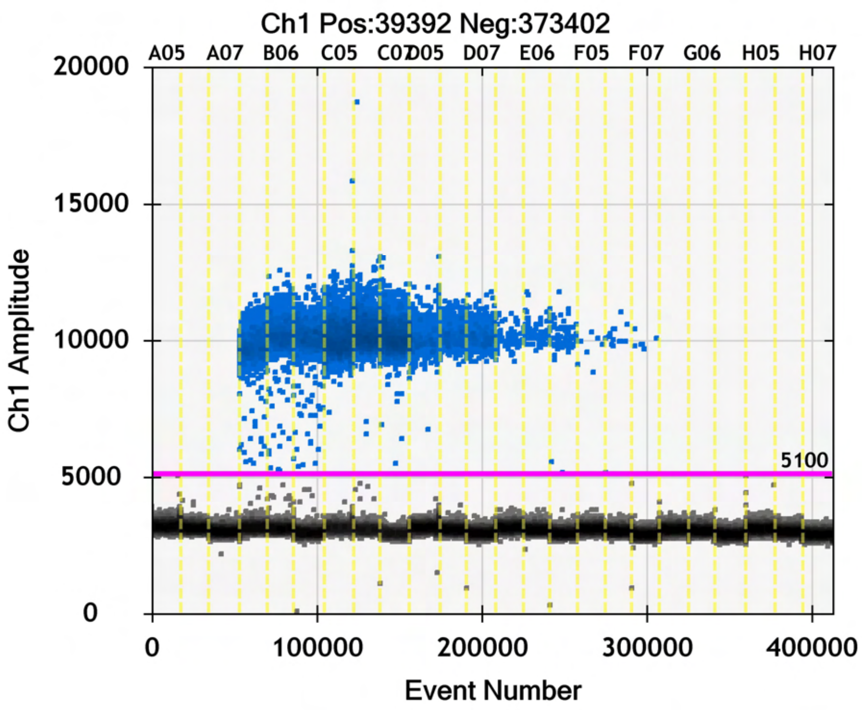

B

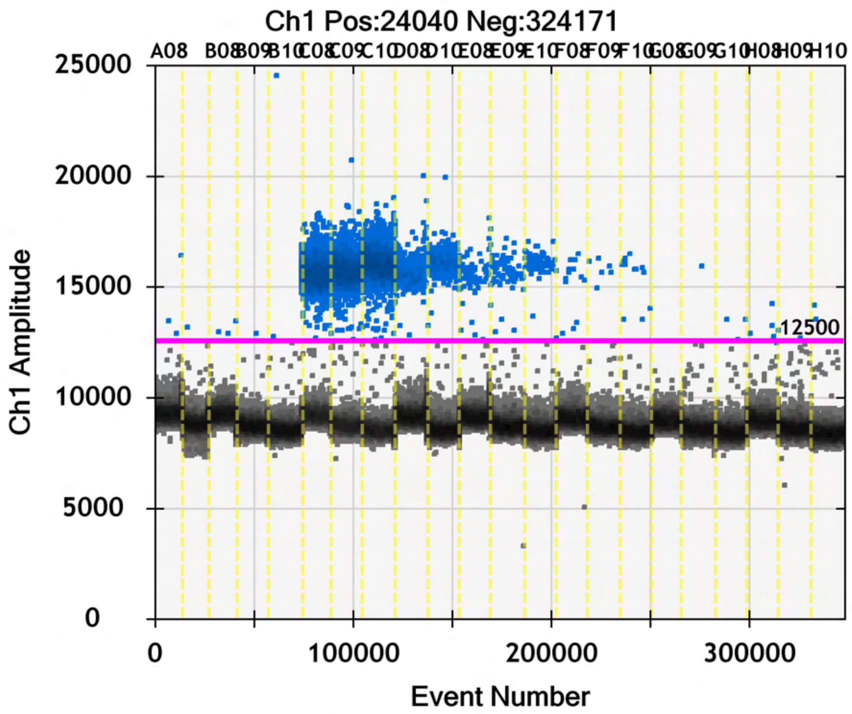

Figure S8. Comparison of ddPCR reactions using NSM_amoAF/R and amoA332F/822R PCR reactions. 1D plots from the bacterial *amoA* ddPCR droplet reads showed good signal separation for the positive droplets from the negative droplets of the reactions using the designed primers (A), but the signal separation between the positive and negative droplets of the reactions using the published primers (72) was poorer and the positive control plasmids failed to amplify (B).

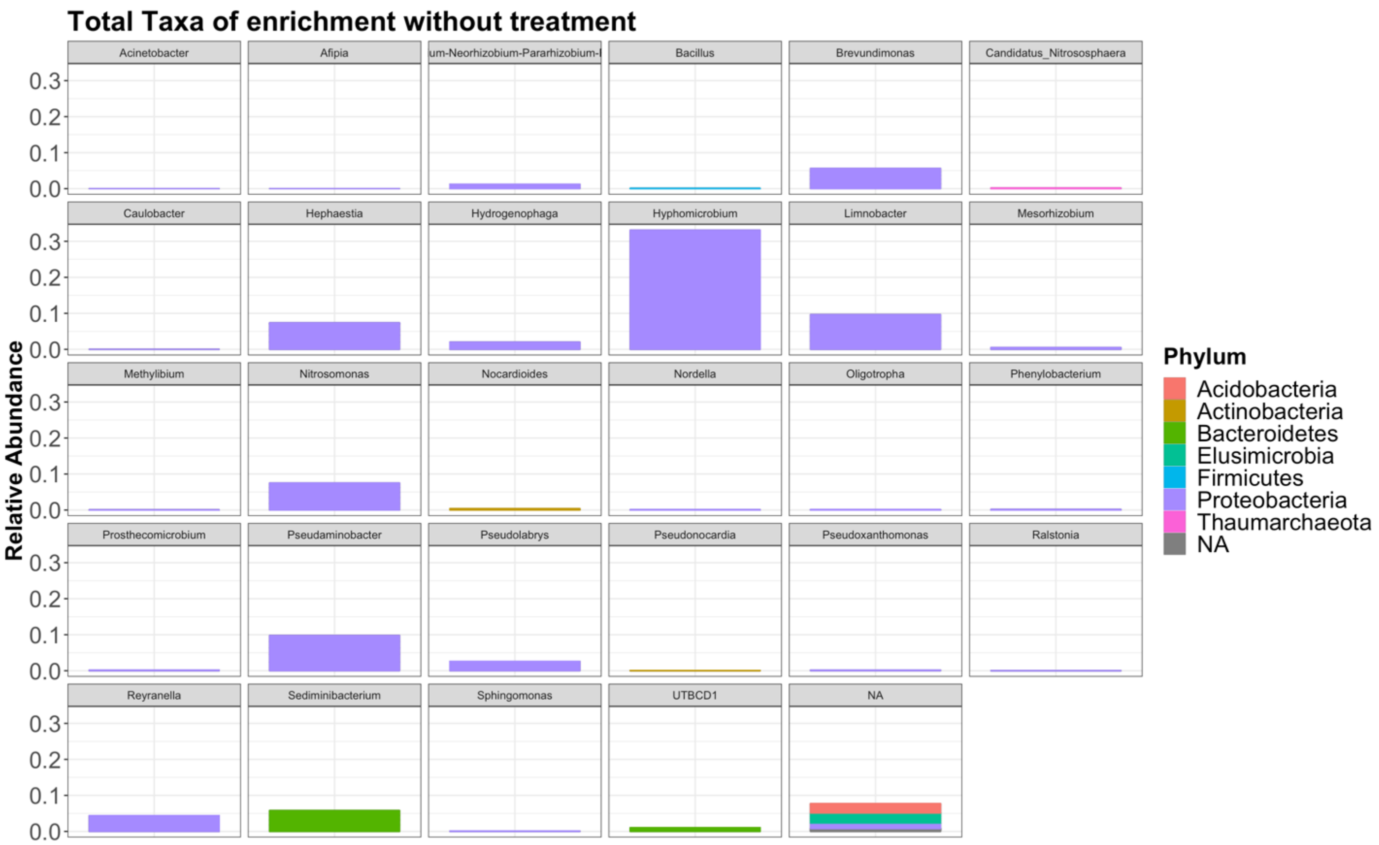

Figure S9. The total taxa sequenced from the enrichment without treatment. The taxa are faceted by genera and colored by phyla. Proteobacteria phylum appeared to be the dominant taxa, with *Hyphomicrobium* being the dominant genus in the enrichment. NA refers to taxa not identifiable to genus level.

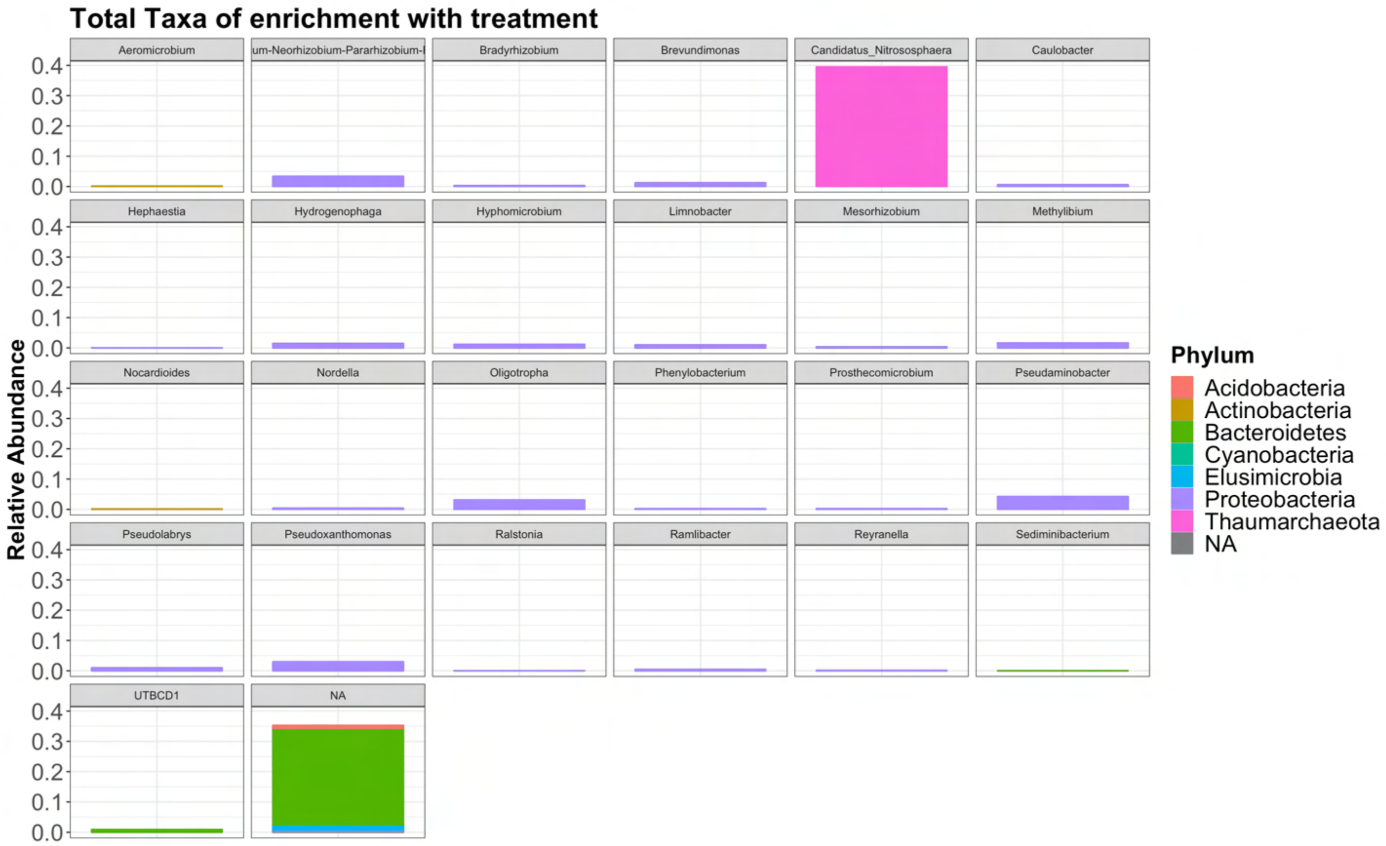

Figure S10. The total taxa sequenced from the enrichment with DMTU and pyruvate treatment. The taxa are faceted by genera and colored by phyla. AOA (Thaumarchaeota) phylum and Bacteroidetes phylum appeared to be the dominant taxa. NA refers to taxa not identifiable to genus level.

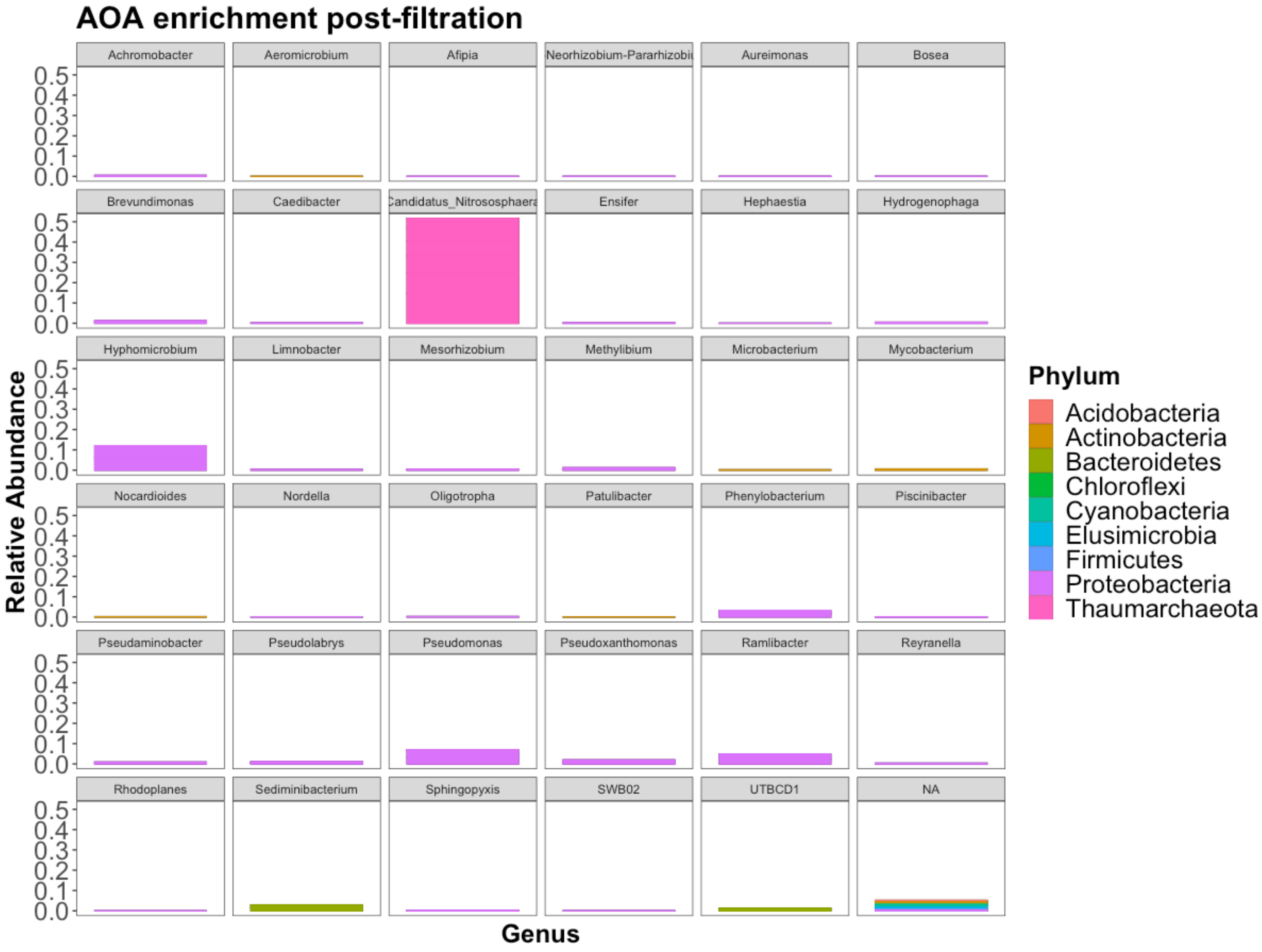

Figure S11. The total taxa sequenced from the enrichment with DMTU and pyruvate treatment and filtration with 0.45 μm membrane filter. The taxa are faceted by genera and colored by phyla. AOA (Thaumarchaeota) phylum and Proteobacteria phylum appeared to be the dominant taxa. NA refers to taxa not identifiable to genus level.

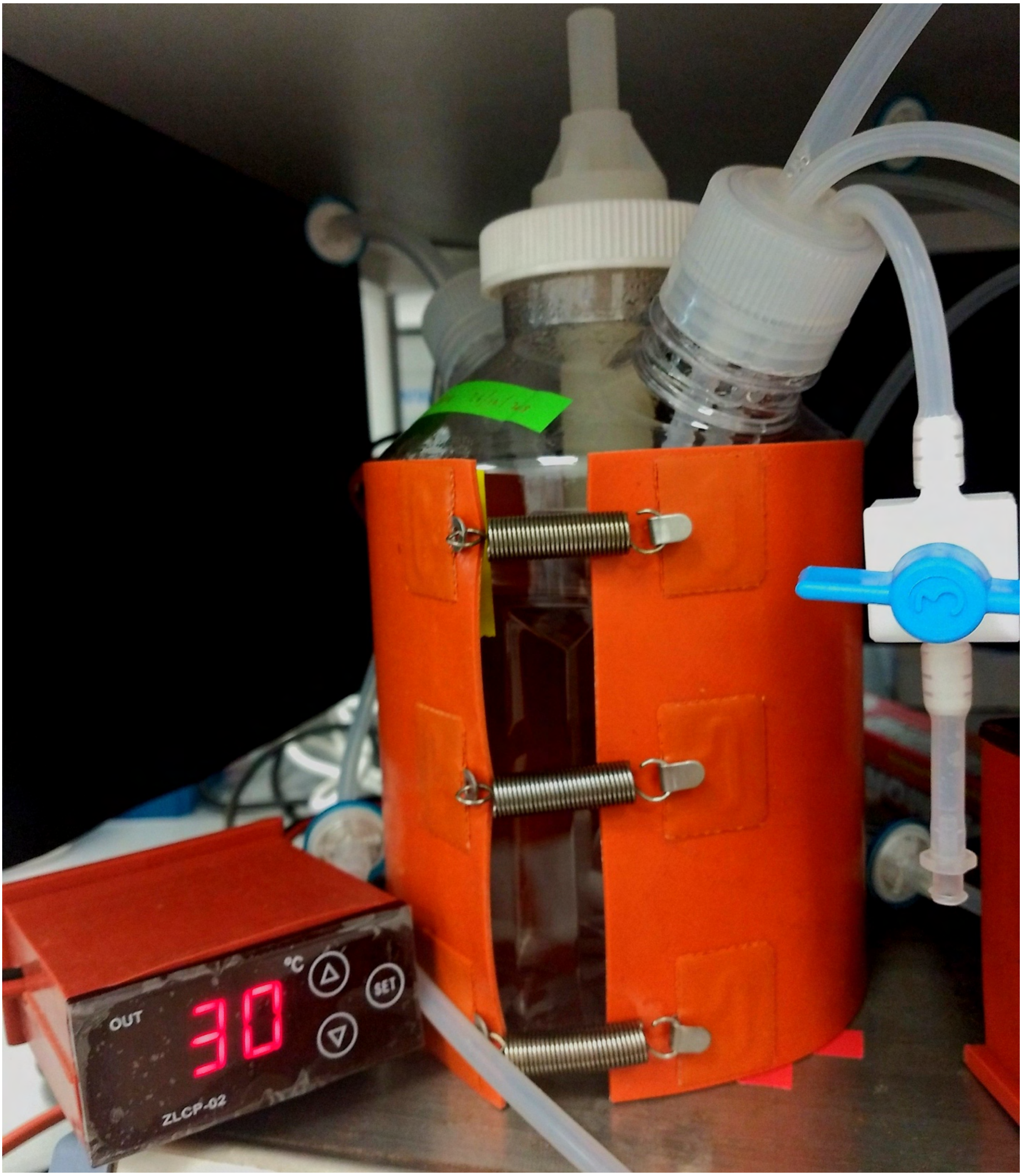

Figure S12. Set up of the enrichment culture vessel: Nalgene™ Polycarbonate Magnetic Culture Vessel; Silicon heating jacket with thermostat; Sampling tube with valve; filtered air supply tube with aeration stone; filtered air vent.

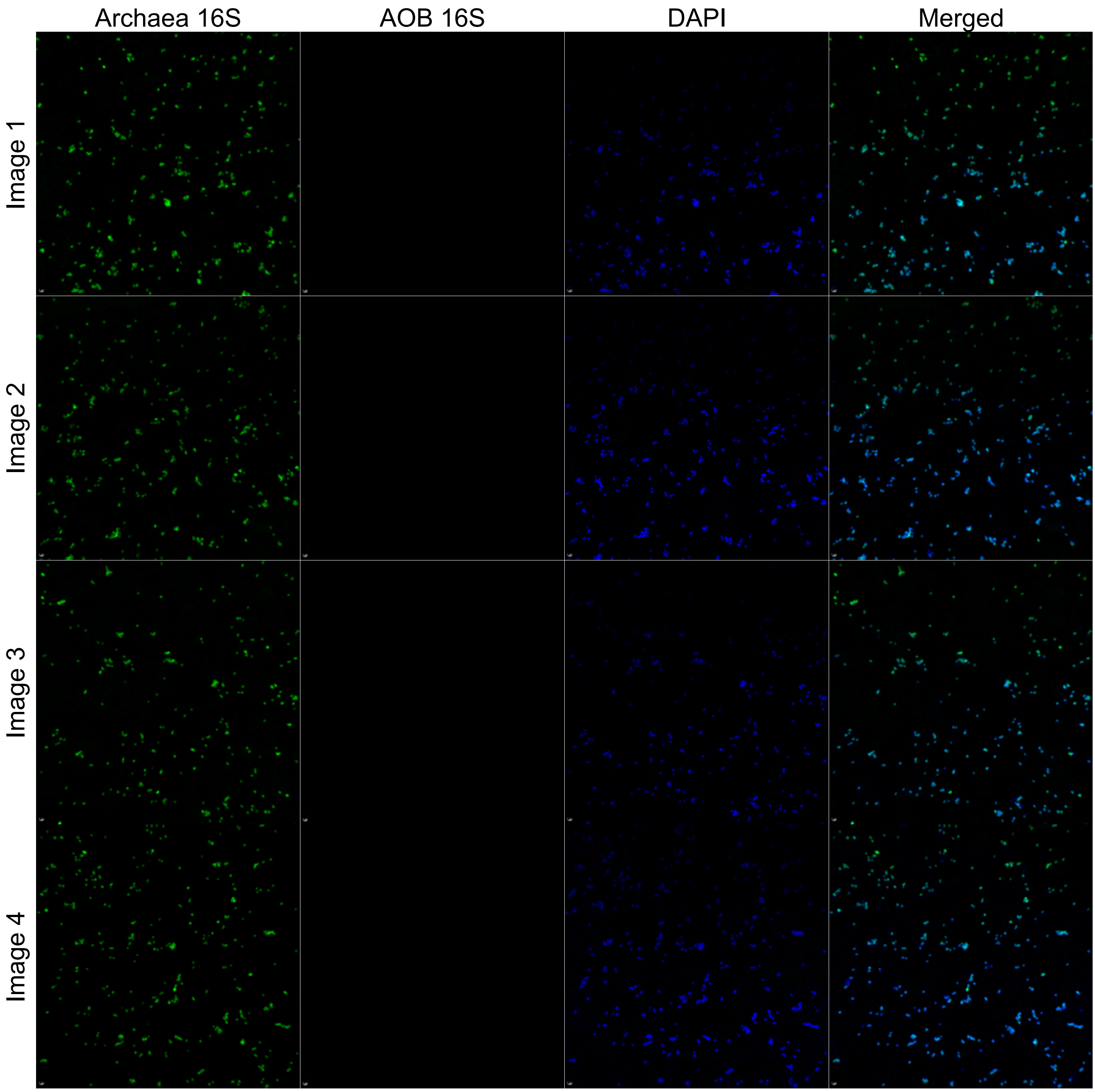

Figure S13. Fluorescent *in-situ* hybridization of the AOA enrichment observed with confocal laser scanning microscopy under 100x objective with oil immersion. The scale bar is at 1 μm. AOB cells were not detected, and total cells were stained with DAPI. The AOA cells have a diameter of approximately < 0.5 μm.

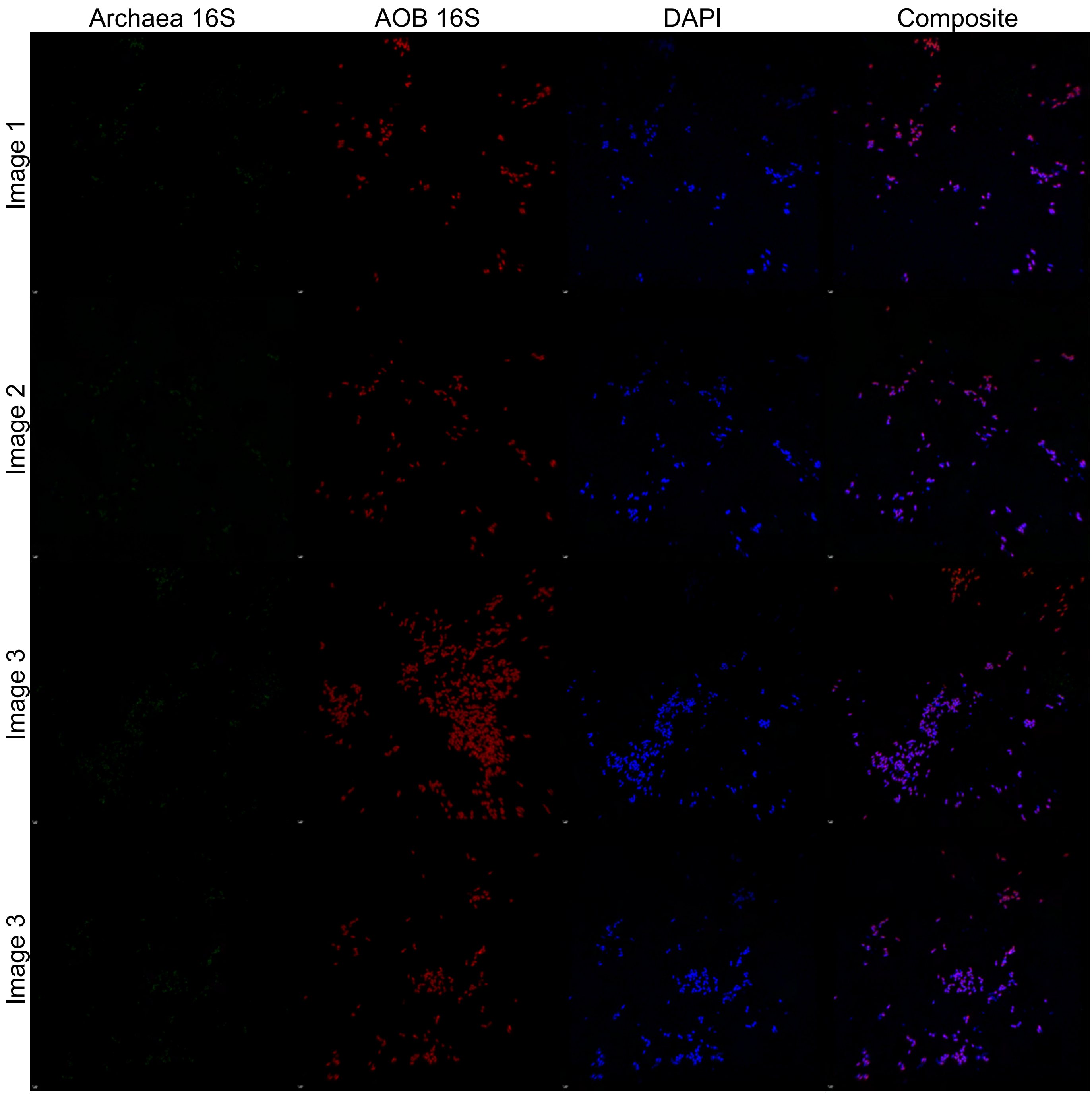

Figure S14. Fluorescent *in-situ* hybridization of the AOB enrichment observed with confocal laser scanning microscopy under 100x objective with oil immersion. The scale bar is at 1 µm. AOA cells were not detected although faint Archaea 16S signals were generated, and total cells were stained with DAPI. The AOB cells are approximately 0.5 – 1 µm long.
